# Salting-out effect promoting highly efficient ambient ammonia synthesis

**DOI:** 10.1038/s41467-021-23360-0

**Published:** 2021-05-27

**Authors:** Mengfan Wang, Sisi Liu, Haoqing Ji, Tingzhou Yang, Tao Qian, Chenglin Yan

**Affiliations:** 1 College of Energy, Key Laboratory of Advanced Carbon Materials and Wearable Energy Technologies of Jiangsu Province, Soochow University, Suzhou, China; 2 School of Chemistry and Chemical Engineering, Nantong University, Nantong, China

**Keywords:** Heterogeneous catalysis, Electrocatalysis

## Abstract

The electroreduction of nitrogen to ammonia offers a promising alternative to the energy-intensive Haber–Bosch process. Unfortunately, the reaction suffers from low activity and selectivity, owing to competing hydrogen evolution and the poor accessibility of nitrogen to the electrocatalyst. Here, we report that deliberately triggering a salting-out effect in a highly concentrated electrolyte can simultaneously tackle the above challenges and achieve highly efficient ammonia synthesis. The solute ions exhibit strong affinity for the surrounding H_2_O molecules, forming a hydration shell and limiting their efficacy as both proton sources and solvents. This not only effectively suppresses hydrogen evolution but also ensures considerable nitrogen flux at the reaction interface via heterogeneous nucleation of the precipitate, thus facilitating the subsequent reduction process in terms of both selectivity and activity. As expected, even when assembled with a metal-free electrocatalyst, a high Faradaic efficiency of 71 ± 1.9% is achieved with this proof-of-concept system.

## Abstract (web summary)

The electroreduction of nitrogen to ammonia offers a promising alternative to the Haber–Bosch process but suffers from low activity and selectivity. Here, authors demonstrate that triggering a salting-out effect in a highly concentrated electrolyte can achieve highly efficient ammonia synthesis.

## Introduction

Considering the great impact of ammonia (NH_3_) on modern society, the efficient activation of dinitrogen (N_2_) is currently among the most important topics^1,2^. In this context, the electrochemical nitrogen reduction reaction (NRR) under ambient conditions, as a sustainable alternative to the century-old Haber–Bosch process, has attracted growing research interest in recent years^3,4^. Despite tremendous efforts, however, efficient NRRs still face great practical challenges. One of the major concerns lies in the poor accessibility of N_2_ molecules to the electrocatalyst, which limits the ammonia yield rate to a lower level^5^. Another significant issue arises from the intense competition with the hydrogen evolution reaction (HER), leaving nitrogen fixation with low Faradaic efficiency^6^. An overwhelming excess of H_2_O molecules are at the heart of electrochemical NRR systems due to their dual roles as the solvent and proton source^7,8^. Therefore, we believe that modification of the aqueous electrolyte may provide a critical advantage in promoting the NRR process in terms of both selectivity and activity.

The salting-out effect is a modern phenomenon, and its basic mechanism involves a change in the solubility of a nonelectrolyte in an aqueous solution with the addition of a salt^9^. Specifically, when solute ions are added into the solution of a nonelectrolyte, there will be competition among them for H_2_O molecules. Since ions are able to attract polar H_2_O molecules via Coulombic interactions, this competition is won by the solute ions, and nonelectrolytes with less affinity for H_2_O molecules lose. The H_2_O molecules preferentially move away from the nonelectrolytes towards the solute ions, forming a hydration shell around the ions. As a result, the H_2_O involved in the hydration of the ions can no longer serve as either a proton source or solvent, not only effectively suppressing the HER but also driving the nonelectrolyte to precipitate from the solution due to decreased dissolution^10^. Considering N_2_ as the nonelectrolyte, the precipitated N_2_ may move around randomly, with no tendency towards nucleation because of the relatively high energy barrier. However, in a heterogeneous catalytic system, when a solid phase is present in the system, the precipitated N_2_ is more inclined to accumulate on the heterogeneous interface due to the lowered nucleation barrier^11^. When the salt concentration is high enough, a high level of hydration can be achieved, delivering abundant N_2_ molecules to the electrocatalyst surface, and greatly facilitating the subsequent adsorption and reaction processes. Bearing these in mind, the salting-out effect can be elegantly applied in electrochemical NRR systems, simultaneously promoting both selectivity and activity.

Here we report a strategy in which the salting-out effect in a highly concentrated electrolyte solution plays a critical role in promoting ambient ammonia synthesis. Simulations suggest both enhanced nitrogen flux and optimized H_2_O diffusibility at the reaction interface, creating an ideal environment for highly selective and active NRR. The deactivation of H_2_O and corresponding suppression of HER, as well as the induced nitrogen enrichment on the electrocatalyst surface, were experimentally verified by in situ observations, affording clear insight into the mechanism of this novel electrochemical system. A superior performance was obtained on a metal-free electrocatalyst in this proof-of-concept system, as expected, in terms of both selectivity and activity, with a Faradaic efficiency of 71 ± 1.9% and a yield rate of (9.5 ± 0.4) × 10^−10^ mol s^−1^ cm^−2^ at −0.3 V vs. reversible hydrogen electrode (RHE), approaching the target set by the U.S. Department of Energy’s ARPA-E REFUEL Program.

## Results

### Theoretical simulations of the heterogeneous systems

**Fig. 1:**
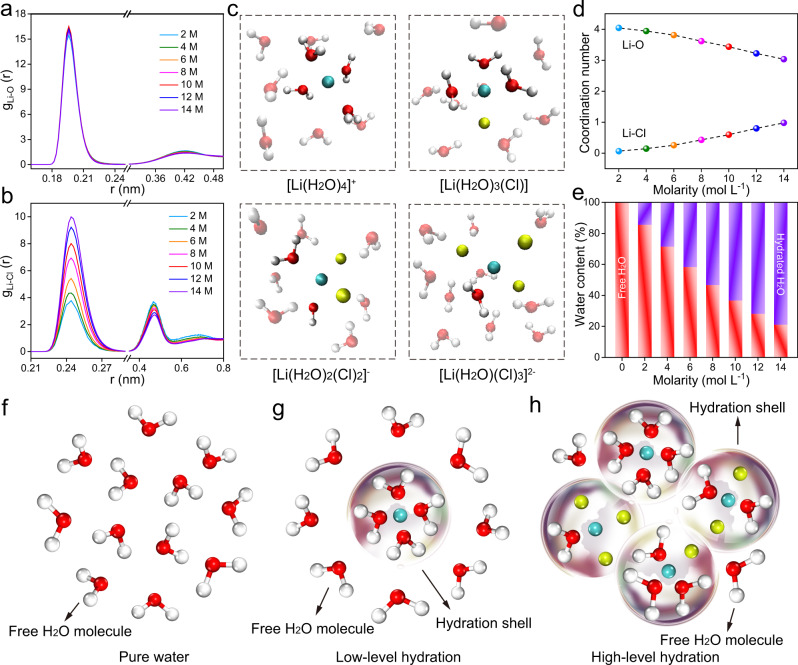
Molecular dynamics simulations of the hydration system. Radial distribution function for **a** Li^+^-O and **b** Li^+^-Cl^−^ interactions. **c** Representation of the four stable clusters for the LiCl coordination sphere. **d** Coordination number of Li^+^-O and Li^+^-Cl^−^ as a function of LiCl concentration. **e** Water content in LiCl solutions of different concentrations. Illustration of the evolution of the hydration shell in **f** pure water, **g** a low-level hydration system, and **h** a high-level hydration system. The cyan, yellow, red, and white spheres represent Li, Cl, O, and H atoms, respectively.

LiCl, with its outstanding solubility in water, can realize high levels of hydration^12^, so it was chosen as the prototype salt for this study. Molecular dynamics (MD) simulations were conducted to probe the structural and dynamic properties of the hydration systems at different LiCl concentrations (2, 4, 6, 8, 10, 12, and 14 M). The Li^+^-H_2_O interactions were first considered, and the radial distribution functions (RDFs) are depicted in Fig. 1a. Known as the structure-maker ion^13^, Li^+^ usually exhibits a strong interaction with surrounding H_2_O molecules due to its small size and high charge density, thus forming a rigid hydration sphere, as indicated by the sharp Li^+^-O peak located at 0.196 nm. The peak at approximately 0.42 nm is rather flat and poorly resolved, so the second hydration sphere of Li^+^ is much less pronounced and is not considered in our work. For the Li^+^‒Cl^−^ interaction, due to the large difference in their ionic radii and the category (Li^+^ as structure-maker ion and Cl^−^ as the structure-breaker ion), ion pairing theoretically seldom occurs, and they usually remain apart in solution^14^. However, clear evidence of ion pairing has been observed by neutron diffraction in previous work^15^. Here, in our simulation, Li^+^‒Cl^−^ interactions were confirmed by the well-defined peak at 0.244 nm in Fig. 1b. This signal intensifies as the salt concentration increases, since more Cl^−^ will penetrate the lithium hydration sphere and replace some of the H_2_O molecules. The lithium hydration sphere maintains a balance between four stable clusters: [Li^+^(H_2_O)_4_]^+^, [Li^+^(H_2_O)_3_Cl^−^], [Li^+^(H_2_O)_2_(Cl^−^)_2_]^−^, and [Li^+^(H_2_O)(Cl^−^)_3_]^2−^, as displayed in the snapshots in Fig. 1c, and the total coordination number of lithium ions is 4 for all cases. Good agreement can be seen by comparing our results with values in the literatures^12,16–19^, demonstrating that the structural properties of hydration systems have been validated.

With increasing salt concentration, although the hydration number of Li^+^ decreases slightly due to the participation of Cl^‒^ (Fig. 1d and Supplementary Fig. 1), the total number of H_2_O molecules of hydration continues to increase, which means a continuous decrease in free H_2_O (Fig. 1e). That is, the proton activity will be influenced by the salt concentration compared with pure water (Fig. 1f). In a low hydration system, a few lithium hydration shells are uniformly distributed in the system, and sufficient free H_2_O molecules will be available (Fig. 1g), so that water electrolysis and sustained hydrogen evolution would still be favoured due to the adequate proton supply. When the concentration of LiCl is further increased, an increasing number of H_2_O molecules take part in the lithium hydration spheres, and only finite free H_2_O is available (Fig. 1h), significantly diminishing the proton supply.

**Fig. 2:**
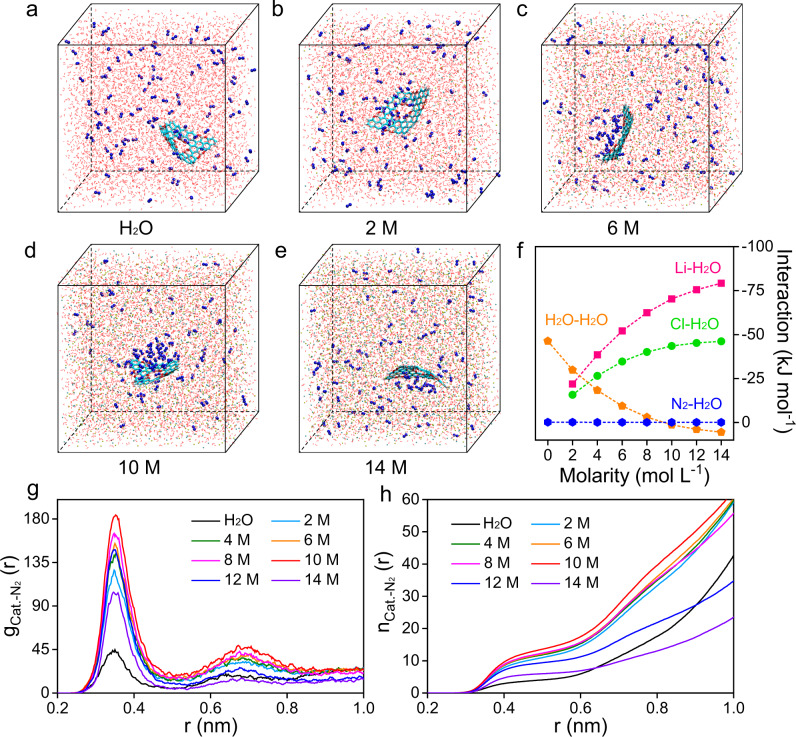
Molecular dynamics simulations of the heterogeneous catalysis systems. **a**–**e** Snapshots of the heterogeneous catalysis systems with different LiCl concentrations at 20 ns. **f** Interaction energies of ion–water, water–water, and gas–water within the heterogeneous catalysis system. **g** Radial distribution function (RDF) and **h** integrated RDF of nitrogen molecules around the catalyst surface.

Based on the above results, LiCl is supposed to be an ideal solute to trigger the salting-out effect. Therefore, the impact of LiCl on the heterogeneous catalysis systems was subsequently investigated. Considering that metal-free materials can show long durability in addition to their intrinsic advantages of low cost and environmental friendliness, boron-decorated carbon was chosen as the model catalyst here (Supplementary Fig. 2). Since nitrogen is continuously fed into the system during the actual experiments, a supersaturated state was used in the simulations. Snapshots of different salt concentrations are shown in Fig. 2a–e. Obviously, there is nitrogen enrichment at the electrolyte/electrocatalyst interface in concentrated solutions compared with that in pure water. Several interactions within the system were calculated to explore the underlying mechanism (Fig. 2f and Supplementary Table 1). In pure water, only H_2_O‒H_2_O interactions and weak N_2_–H_2_O interactions exist. The nitrogen molecules maintain a state of dynamic equilibrium and are uniformly distributed in the hydrogen bond network with no localized concentration difference. In LiCl solution, Li^+^, Cl^−^, and N_2_ molecules compete with each other for H_2_O molecules. Given the tiny quadrupole moment of N_2_, it always exhibits the lowest affinity for H_2_O molecules. On the other hand, both Li^+^‒H_2_O interactions and Cl^−^‒H_2_O interactions become more intense from 2 to 14 M LiCl solutions, which is consistent with previous studies^19,20^. With increasing salt concentration, the ions gradually disrupt the hydrogen bond network and prevent the closest water molecules from being tetrahedrally oriented. Along with the decrease in hydrogen bonds, namely, the decrease in the overall H_2_O‒H_2_O interaction in the whole system^19,20^, the density of immobilized H_2_O molecules around the ions continues to increase. Consequently, the density of available free water around the nitrogen molecules significantly decreases, forcing them to precipitate from the solution and gradually accumulate on the electrolyte/electrocatalyst interface due to the lower nucleation barrier. Taking 10 M LiCl as the example, in great contrast to the system without an electrocatalyst, where the N_2_ molecules are uniformly distributed all the time (Supplementary Fig. 3), obvious nitrogen enrichment can be observed at the electrocatalyst surface (Supplementary Fig. 4) due to van der Waals (vdW) interactions between them (Supplementary Fig. 5). To quantitatively demonstrate the N_2_ accumulation effect, the RDF and the integrated RDF were calculated (Fig. 2g, h). Clearly, a much higher density of N_2_ molecules around the electrocatalyst can be observed in highly concentrated electrolyte solutions compared with pure water within the same distance. This N_2_ accumulation effect reached its peak in 10 M LiCl, which is in great accordance with the snapshots, indicating that this concentration has the greatest potential to benefit the subsequent NRR process.

Notably, the nitrogen enrichment gradually declines for higher concentrations beyond 10 M. At that concentration, the hydrogen bond network is already so disrupted that, even when the concentration increases further, the effects on the hydrogen bonding of the water molecules are minor^21^. On the other hand, the diffusion of the nitrogen molecules would be significantly inhibited by the rigid hydration shells throughout the system, limiting their access to the heterogeneous interface. To demonstrate this, the diffusion coefficients of H_2_O and N_2_ were calculated from the mean square displacement (Supplementary Fig. 6). They are shown in Supplementary Fig. 7 as a function of the salt concentration, and the values were close to those in previous studies^19,20,22,23^. As expected, the diffusion coefficients of H_2_O and N_2_ both decrease to ultralow values in solutions at extremely high concentrations. That is, when the salt concentration is high enough, most H_2_O molecules are involved in the lithium hydration spheres and contribute substantially to a dense system, greatly slowing down the diffusion of nitrogen. Therefore, the much lower nitrogen accumulation in 12 and 14 M LiCl compared with 10 M LiCl is well explained. With such electrolytes, the systems are anticipated to deliver unsatisfactory NRR performance due to the limited nitrogen supply to the electrolyte/electrocatalyst interface.

To verify this phenomenon from a more macroscopic standpoint, finite element simulations using the COMSOL Multiphysics software were conducted. The modelling of the heterogeneous catalysis system is shown in Supplementary Fig. 8. Pure N_2_ was continuously fed into the system at a steady flow rate of 30 sccm, and two-dimensional contour plots of the N_2_ volume fraction after 100 s were extracted to evaluate its accumulation on the electrocatalyst surface. As expected, a thick layer of nitrogen was clearly observed around the electrocatalyst in 10 M LiCl with respect to pure water, which is in excellent agreement with the MD simulations (Supplementary Fig. 9). Moreover, in such an aggregation layer, once the nitrogen content at one point is below a certain value, it will be immediately replenished by the salted-out nitrogen from the surroundings. Therefore, nitrogen enrichment is maintained at a steady state and can eliminate the influence of nitrogen consumption in the actual NRR process. Based on the above results, 10 M LiCl should achieve a perfect balance between the nitrogen supply and proton activity within the Stern layer, making it an ideal system for highly efficient NRR.

### Physicochemical characterization of the LiCl solution

Physicochemical characterization of the LiCl solutions at different concentrations was performed to verify the theoretical conclusions. The viscosity of the electrolytes, which describes resistance to flow, increases with increasing salt concentration (Supplementary Fig. 10a). The higher the solution concentration, the more important the role of the lithium ion is, and thus the slower the mobility of the solution^24^. The ionic conductivity of the electrolytes initially increases, reaches a maximum at 6 M, and then decreases with further increased concentration (Supplementary Fig. 10b). In principle, the ionic conductivity is proportional to the number of charge carriers and their mobility. However, at higher salt concentrations, an increasing number of charge carriers would induce stronger ion pairing, decreasing the ion mobility and thus the ionic conductivity^25^. A Walden plot of log (molar conductivity, Λ) vs. log (reciprocal viscosity, *η*^−1^)^26^ is further shown in Supplementary Fig. 11. All the points lie slightly below the reference line based on the properties of dilute aqueous KCl solution. This indicates that their molar conductivity is lower than that expected from the fluidity, suggesting their intermediate character between an ionic liquid and an aqueous solution, which is clear evidence of the existence of ion pairing^27^. The interplay among Li^+^, Cl^−^, and H_2_O was also investigated with various spectroscopic techniques. In pure water, the O-H stretching vibration gave rise to a broad band in both Raman and Fourier transform infrared (FTIR) spectra (Supplementary Fig. 12), corresponding to various water molecules with different hydrogen-bonding environments in water clusters^28,29^. With increasing salt concentration, an increasing number of water clusters participate in the lithium hydration shell, and the hydrogen bond network is dramatically disrupted. As a result, a sharp O-H vibration peak appeared at the expense of the broad water-cluster band, explaining the greatly reduced proton activity in highly concentrated LiCl solutions. Since the water ^17^O signal is rather sensitive to the presence of salt, ^17^O nuclear magnetic resonance (NMR) measurements were further performed to better understand the behaviour of H_2_O molecules in LiCl solutions (Supplementary Fig. 13). As the salt concentration increases, the chemical shift of the characteristic peak steadily decreases accompanied by peak broadening. The downfield shift in the peak originates from the direct depletion of the lone-pair electrons on water O by the lithium ion, which deshields the O nucleus^30^, and the peak broadening can be attributed to the decreasing *T*_2_ relaxation time derived from the increased viscosity^31^. Subsequently, the water activity^32^ of the LiCl solution was measured to probe the availability of free water at the macroscopic level (Supplementary Table 2). Higher salt concentrations resulted in lower water activities, and thus fewer free water molecules available to serve as proton donors. Moreover, the concomitant salt hydration polarization can be evaluated based on the contact angles between the substrate and the solution droplets with respect to pure water^33^. The extent of the polarization is increased with increasing salt concentration based on the increased contact angle (Supplementary Fig. 14), corresponding to the positively correlated hydration level. The results of the physicochemical characterization matched well with the MD simulations, and we can anticipate that, in a high hydration system, H_2_O molecules should be severely confined within the lithium hydration shells, so that the electron-stealing HER should be greatly suppressed.

### HER performance and in situ characterization

The electrochemical experiments were subsequently conducted with LiCl solutions at different concentrations as the electrolyte. Covalent organic frameworks (COFs) with abundant boron sites^34^ were chosen as the boron source for the proof-of-concept electrocatalyst. They were uniformly grown on a nanosheet substrate (graphene oxide) to achieve great exposure of active sites (B-COF/GO, Supplementary Fig. 15) and then appropriately pyrolyzed to ensure conductivity. Single-atom boron-decorated carbon was obtained and used for the following electrochemical measurements (SAB/C, Supplementary Fig. 16).

**Fig. 3:**
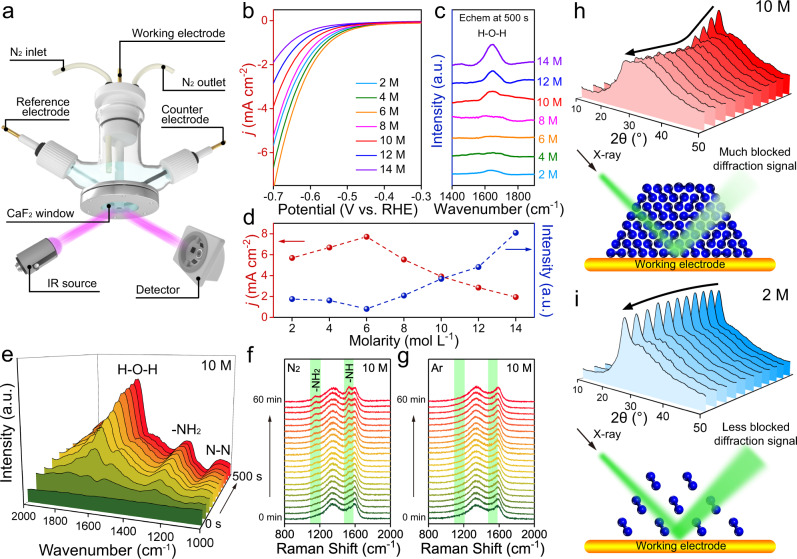
HER performance and in situ characterization. **a** Schematic of the tailor-made electrolytic cell for in situ Fourier transform infrared (FTIR) characterization. **b** Linear sweep voltammetry (LSV) curves of different systems under an Ar-saturated environment. **c** In situ FTIR spectra captured under electrochemical (Echem) reaction conditions at −0.3 V vs. RHE at 500 s. a.u., arbitrary units. **d** Comparison of the hydrogen evolution reaction (HER) activities and intensities of the H-O-H bending vibration in the FTIR spectra as a function of salt concentration. **e** In situ FTIR spectra with 10 M LiCl as the electrolyte at −0.3 V vs. RHE as a function of time. In situ Raman spectra with **f** N_2_-saturated and **g** Ar-saturated 10 M LiCl as the electrolyte at −0.3 V vs. RHE as a function of time. In situ X-ray diffraction (XRD) contour plots of working electrodes and the corresponding schematic of the mechanism with **h** 10 M and **i** 2 M LiCl as electrolytes during the nitrogen reduction reaction (NRR) process at −0.3 V vs. RHE as a function of time.

To explore whether the suppression of HER works in the practical catalytic process, in situ FTIR characterizations of different systems were performed in a tailor-made device (Fig. 3a). Linear sweep voltammograms (LSVs) were first measured under an Ar-saturated environment to evaluate the HER performance of the different electrocatalytic systems (Fig. 3b). Interestingly, the HER activity increases slightly from 2 to 6 M LiCl and then decreases from 8 to 14 M LiCl. The corresponding in situ FTIR spectra show that the intensity of the H-O-H bending vibration of H_2_O molecules presents the opposite trend as the HER activity (Fig. 3c, d). This phenomenon agrees very well with the experimental results. From 2 to 6 M LiCl, an increasing number of H_2_O molecules around the working electrode were electrolyzed, leaving fewer free molecules to be captured by FTIR spectroscopy. As the salt concentration was further increased, the HER was gradually suppressed, and more H_2_O molecules remained at the electrode surface, explaining the increase in the intensity of the H-O-H bending vibration from 8 to 14 M LiCl. By combining the electrochemical measurements and in situ characterization, the highly concentrated LiCl solution is confirmed to be an efficient suppressor of the HER.

The whole process of the efficient NRR process was then observed by a combination of various in situ techniques. A 10 M LiCl solution was chosen as the electrolyte as it offered the greatest nitrogen accumulation within the Stern layer, as indicated by the MD simulations and finite element simulations. For in situ FTIR characterizations as a function of time, in addition to the H-O-H bending mode of the water molecules at 1669 cm^−1^, two peaks at 1298 and 1090 cm^−1^, attributed to the -NH_2_ wagging and N-N stretching of adsorbed N_2_H_*y*_ species, respectively, were also monitored (Fig. 3e)^35^. In situ Raman spectra were further captured, and two peaks located at 1152 and 1526 cm^−1^, corresponding to -NH_2_ and -NH, respectively, appear and increase in intensity in the N_2_-saturated environment (Fig. 3f)^36^, whereas no such phenomenon was observed in the Ar-saturated environment (Fig. 3g).

To further verify the influence of the salting-out effect on nitrogen adsorption on the working electrode, in situ X-ray diffraction (XRD) characterizations of different LiCl concentrations were conducted. For 10 M LiCl (Fig. 3h), the major carbon diffraction located at approximately 26.5° is observed under the original conditions of the working electrode and gradually weakens in the first half hour. This can be attributed to the continuous nitrogen adsorption because it partially blocks the carbon signal during the XRD characterization. As the reaction proceeds, the adsorption and consumption of nitrogen reach a balance, and the carbon signal remains steady over the next half hour. In great contrast, when using 2 M LiCl solution as the electrolyte (Fig. 3i), since it is much less able to induce nitrogen enrichment at the electrode surface, less intense changes in the carbon peak are observed in the XRD patterns for a full hour. Therefore, it should be noted that the salt concentration should be sufficiently high to realize the desired nitrogen accumulation via the salting-out effect. The dilute solution only deposits a small amount of nitrogen at the electrode surface, which is not sufficient for achieving an efficient NRR process. The above observations are in excellent agreement with the theoretical results and afford clear insight into the mechanism of our novel electrochemical system. Since it can simultaneously suppress the HER as well as induce nitrogen enrichment within the Stern layer, a highly concentrated LiCl solution is supposed to serve as an ideal electrolyte for highly selective NRR.

### Electrocatalytic nitrogen reduction performance

**Fig. 4:**
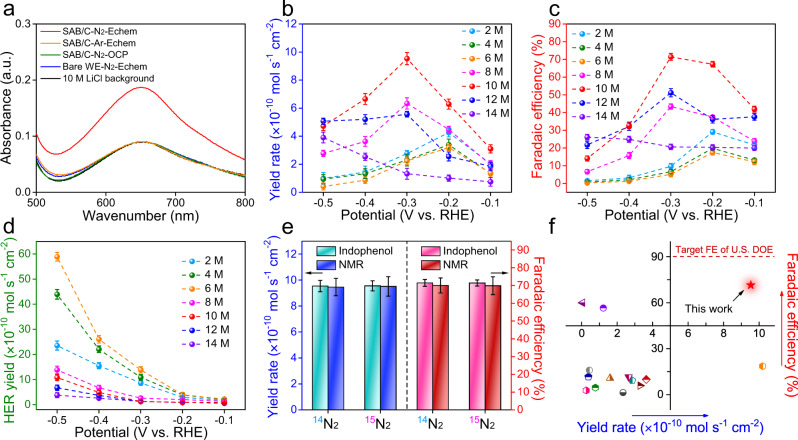
Electroreduction of N_2_ to NH_3_ under ambient conditions. **a** Ultraviolet-visible absorption spectra of the electrolytes under different conditions. No apparent NH_3_ was detected for the control experiments with Ar-saturated electrolyte under Echem, N_2_-saturated electrolyte under open circuit potential (OCP), or bare working electrode (WE) without single-atom boron-decorated carbon catalyst (SAB/C) in N_2_-saturated electrolyte under Echem. **b** NH_3_ yield rates, **c** corresponding Faradaic efficiencies, and **d** HER yield rates in different systems. **e** Comparison of the Faradaic efficiency and NH_3_ yield rate using different feeding gases for NRR at −0.3 V vs. RHE either by indophenol or nuclear magnetic resonance (NMR) method. The error bars correspond to the standard deviations of measurements of three separately prepared samples under the same conditions. **f** Comparison of our results with state-of-the-art noble-metal-free electrocatalysts in terms of yield and Faradaic efficiency (FE); detailed data are provided in Supplementary Table 4. The Faradaic efficiency of our work is very close to the target set by the U.S. Department of Energy (DOE)’s ARPA-E REFUEL Program.

The electrocatalytic NRR measurements were performed using a gas-tight H-type cell separated by a Celgard membrane with hydrophilic treatment^37^. An in-line acid trap of the cathode chamber filled with 0.1 M HCl was set to avoid volatilization of the produced ammonia^38,39^. A rigorous experimental protocol was strictly followed to achieve reliable proof of the NRR (Supplementary Fig. 17)^37^. Before electrochemical measurements, the presence of NH_3_ or NO_*x*_ in the feeding gas was completely avoided by sufficient purification of the feed N_2_ (Supplementary Figs. 18 and 19)^40^. Subsequently, control experiments were performed to further exclude adventitious contamination, such as ammonia from atmosphere or human breath, trace on the equipment, or residues in the electrocatalyst. No ammonia could be detected in the electrolyte saturated with Ar under the electrochemical conditions or with N_2_ but under open circuit potential either by a colorimetric or NMR method (Supplementary Figs. 20 and 21). In contrast, with N_2_ under the electrochemical tests, the accumulated ammonia significantly exceeds the background (Fig. 4a), so its synthesis was confirmed to exclusively originate from the electroreduction of dinitrogen.

The quantitative analysis of the NRR performance of different systems was systematically conducted through chronoamperometry measurements (Supplementary Fig. 22). The produced ammonia and the possible by-product hydrazine were spectrophotometrically evaluated (Supplementary Figs. 23 and 24), whereas only ammonia was detected in this work (Supplementary Table 3). The mean value of the NRR current density, the average NH_3_ yields, and the corresponding Faradaic efficiencies at potential ranges from −0.1 to −0.5 V vs. RHE are compared in Supplementary Fig. 25 and Fig. 4b, c. As suggested by the LSV curves in Fig. 3b, the HER activity in the system increases slightly in low-concentration LiCl electrolytes, such that most protons and electrons are involved in hydrogen evolution. As a result, the ammonia yield rate and the Faradaic efficiency remain low and decrease from 2 to 6 M LiCl. With further increasing salt concentration, the HER activity is gradually suppressed, and the protons are more inclined to attack the adsorbed nitrogen to proceed with its hydrogenation. Impressively, in 10 M LiCl, the Faradaic efficiency and NH_3_ yield rate both reach their maxima of 71 ± 1.9% and (9.5 ± 0.4) × 10^−10^ mol s^−1^ cm^−2^, respectively, at −0.3 V vs. RHE. The produced ammonia was also quantified using isotope-specific NMR considering that the integrated peak area can be directly related to the content^41^. The standard curve based on NMR spectroscopy is shown in Supplementary Fig. 26, and the calculated ammonia yield is similar to that obtained by the colorimetric method (Supplementary Fig. 27). For 12 and 14 M LiCl, although the HER is further suppressed, nitrogen transfer is inhibited due to the high viscosity, causing the ammonia yield and the Faradaic efficiency to decrease to a certain extent. Hydrogen evolution analyses were also conducted (Supplementary Figs. 28 and 29), and the HER yield rate followed the same trend as the electrochemical measurements (Fig. 4d). More definite proof that the produced ammonia comes from the electrochemical NRR process was obtained from isotope labelling experiments with purified ^15^N_2_ (Supplementary Fig. 30) as the feed gas^42^. No ammonia was detected at the open circuit potential, while at −0.3 V vs. RHE, evident ammonia signal can be captured under the electrochemical tests (Supplementary Figs. 31 and 32). Subsequently, the ^1^H NMR spectra and the corresponding integrated areas of the ^14^NH_4_^+^/^15^NH_4_^+^ peaks as a function of charge passed were captured (Supplementary Figs. 33–35), and the concentration of ^15^NH_4_^+^ was in quantitative agreement with the concentration of ^14^NH_4_^+^ produced under the equivalent conditions (Supplementary Fig. 36). Moreover, 1:1 agreement between the amounts of ammonia and the corresponding Faradaic efficiencies measured in the ^14^N_2_ and ^15^N_2_ reduction tests was obtained both by the colorimetric and NMR method (Supplementary Fig. 37 and Fig. 4e), indicating reliable experimental results. In addition, robust stability is observed in this electrochemical system, as excellent performance can be maintained for ten successive cycles (Supplementary Fig. 38), with the physical characteristics of the catalyst remaining unchanged (Supplementary Fig. 39).

To highlight the critical role of the 10 M LiCl, the NRR performances of SAB/C in traditional electrolytes, including 0.1 M HCl for acid, 0.1 M Na_2_SO_4_ for neutral, and 0.1 M KOH for alkaline, were measured and compared (Supplementary Figs. 40–42). An obvious advantage can be seen in 10 M LiCl, as suggested by the much better performance at all given potentials. Even compared with the state-of-the-art noble-metal-free electrocatalysts, this system still offers superior performance (Supplementary Table 4), and notably, the Faradaic efficiency of 71 ± 1.9% is very close to the target set by the U.S. Department of Energy (DOE)’s ARPA-E REFUEL Program (Fig. 4f)^1^. Considering that only a metal-free material was used as the electrocatalyst in our work, our strategy to simultaneously promote NRR selectivity and activity can be of great scientific and technical impact for efficient and sustainable ammonia synthesis.

## Discussion

In summary, we highlight the importance of the salting-out effect to achieve a highly efficient NRR process, representing a successful strategy for simultaneously promoting NRR selectivity and activity under ambient conditions. In contrast to dilute solutions, where the hydration shell is finite and the amount of free water remains high, highly concentrated salt solutions could break or distort hydrogen bond networks, greatly increasing the density of immobilized H_2_O molecules around the solute ions. Consequently, the density of available free water around the nitrogen molecules significantly decreases, forcing them to precipitate from the solution and gradually accumulate on the electrolyte/electrocatalyst interface. Therefore, with a highly concentrated LiCl solution as the electrolyte, ideal nitrogen enrichment as well as an appropriate proton supply can be achieved within the Stern layer, as suggested by MD simulations and finite element simulations, and verified by various in situ characterizations. As expected, with 10 M LiCl as the electrolyte, even when using a metal-free electrocatalyst, a superior Faradaic efficiency of 71 ± 1.9% and yield rate of (9.5 ± 0.4) × 10^−10^ mol s^−1^ cm^−2^ at −0.3 V vs. RHE can be obtained, outperforming the state-of-the-arts and approaching the target set by the DOE’s ARPA-E REFUEL Program. Our strategy may find wider applicability in electrochemical systems where HER should be appropriately suppressed while the feeding gas should be enriched at the reaction interface.

## Methods

### Computational method and model

MD simulations were carried out with the open-source software Gromacs (version 5.0.5). During model construction, one catalyst model and 100 N_2_ molecules were randomly dispersed in a LiCl solution (0–14 M), which consisted of 0–1400 Li^+^/Cl^−^ ions in 5500 water molecules.

For water, N_2_, and LiCl, the SPC/E model^43^, TraPPE rigid model^44^, and parameters from Chowdhuri et al.^45^ were employed, respectively (Supplementary Table 5). For the catalyst model, the bonded and nonbonded interaction parameters were obtained from the Universal Force Field^46^ by the OBGMX program^47^ (Supplementary Data 1). To generate the partial charges, the initial molecular geometry was optimized at the DFT/B3LYP level of theory with the 6–311 G (d, p) basis set in Gaussian 09 software, which was then analysed by the Multiwfn program^48^ to generate RESP charges for use in MD simulations.

vdW interactions were described by the Lennard Jones (LJ) potential^49^, which was truncated at 1.2 nm. The LJ interaction parameters between unlike atom pairs were generated by the standard Lorentz–Berthelot combination rule^50,51^. Electrostatic interactions were calculated with the particle mesh Ewald method, with the short-range part truncated at 1.2 nm and the long-range part calculated in the reciprocal space with a Fourier spacing of 0.12 nm. The equations of motion were integrated by the leapfrog algorithm with a time step of 1 fs. The intermolecular interactions are expressed as the sum of the vdW interactions described by the LJ potential and the Coulombic interactions^17,24^:

1 $${U}_{ij}=4\times {\varepsilon }_{ij}\times [{({\sigma }_{ij}/{r}_{ij})}^{12}\mbox{-}{({\sigma }_{ij}/{r}_{ij})}^{6}]+({q}_{i}\times {q}_{j}/{r}_{ij})$$

where *r*_*ij*_ is the distance between particles *i* and *j*, *σ* and *ε* are the size parameter and energy parameter, respectively, and *q*_*i*_ is the charge of the *i*th atom (or ion). The potential parameters for unlike site pairs are expressed via the LB mixing rules:

2 $${\sigma }_{ij}=({\sigma }_{i}+{\sigma }_{j})/2$$

3 $${\varepsilon }_{ij}={({\varepsilon }_{i}\times {\varepsilon }_{j})}^{1/2}$$

Initial configurations were energy minimized by the steepest descent algorithm, followed by 50-ps equilibration at 298 K and 1 bar. Then, 20-ns MD runs were performed under the NPT ensemble (298 K, 1 bar). The system temperature and pressure were controlled with a Nose–Hoover thermostat and Parrinello–Rahman barostat, respectively. Three-dimensional periodic boundary conditions were applied throughout simulations. To evaluate the N_2_ accumulation around the catalyst model, the integrated RDFs were calculated as follows:

4 $${n}_{ji}(r){=4\times {\rm{\pi }}\times {\rm{\rho }}}_{j}\times {\int _0}^{r}{g}_{ji}(r){r}^{2}{\rm{d}}r$$

where *g*_*ji*_ is the RDF of *j* about *i*, *ρ*_*j*_ is the bulk number density of *j*, and *r* is the intercept radius.

The finite element simulations were performed by the COMSOL Multiphysics software coupled with the chemical species transport module. The size of the box was 25 × 25 mm. The working electrode was placed at the middle of the box. The N_2_ flow rate was 30 sccm. The electrolytes were H_2_O and 2, 6, 10, and 14 M LiCl solutions.

### Physicochemical characterization of the LiCl electrolyte

Aqueous LiCl solutions at different concentrations (2, 4, 6, 8, 10, 12, and 14 M) were used as the electrolytes, and their pH was adjusted to 7.0 prior to use. The viscosity was measured with a rotational rheometer (Kinexus pro, Malvern, England). The conductivity was measured with a conductivity meter (S230/731-ISM, METTLER TOLEDO). The structure of the LiCl solution was characterized by FTIR spectroscopy (Tensor 27, Bruker), Raman spectroscopy (HR evolution, Horiba Jobin Yvon, France), and ^17^O NMR spectroscopy (Agilent 600 MHz). The water activity was determined using an AW Sprint water activity meter (Novasina, Lachen, Switzerland). The contact angles between the substrate and the droplets of LiCl solutions at different concentrations were measured with a contact angle meter (OCA15, Dataphysics, Germany).

### Catalyst synthesis

Graphite oxide was prepared via the modified Hummers method. To prepare B-COF/GO, 100 mg of GO was sonicated in 20 ml of methanol, and then 300 mg of benzene-1,4-diboronic acid was added. The solution was transferred to a stainless-steel reactor and sealed under nitrogen gas. The reaction was maintained at 90 °C for 24 h, and the product was thoroughly washed with methanol and mesitylene/dioxane solution. The obtained materials were directly dispersed in 20 ml of mesitylene/dioxane solution with 500 mg of benzene-1,4-diboronic acid. The solution was kept at 120 °C for 72 h, and B-COF/GO was obtained by thoroughly washing the product with acetone. The SAB/C was prepared by thermally treating B-COF/GO at 800 °C in a tube furnace for 2 h under an Ar/H_2_ atmosphere.

### Physical characterization of the catalyst

The field emission transmission electron microscopy images were recorded with a Tecnai G2 F20 (FEI, USA). The XRD patterns were acquired on a Bruker D8 Advance (Germany). The FTIR spectra were recorded with a Bruker Tensor 27 (Germany). NMR spectra were acquired with an Agilent 600 MHz instrument (USA). The Raman spectra were recorded on a Horiba Jobin Yvon HR evolution (France). X-ray photoelectron spectroscopic analysis was performed on Kratos Axis Ultra Dld (UK). N_2_-sorption analysis was performed with a surface area and porosimetry instrument (Micromeritics, ASAP 2020, USA).

### Cathode preparation

To prepare the working electrode, 0.25 mg of catalyst was dispersed in a mixture of ethanol and Nafion solution and sonicated for several hours to obtain a homogeneous ink. The homogeneous ink was loaded onto carbon paper with an area of 0.5 × 0.5 cm^2^ and dried under ambient conditions prior to use.

### Nitrogen purification

The ^14^N_2_ commercially purchased from Messer Gas Product Co., Ltd. (Germany, Supplementary Table 6) and ^15^N_2_ commercially purchased from Newradar Special Gas Co., Ltd. (99 atom% ^15^N, Wuhan, China) were successively flowed through acid and alkaline traps to remove possible contaminants. Before being supplied into the electrocatalytic cell, the feed gas was flowed through a drying tube to block water vapour. The ammonia produced from the impurity contribution in the feed gas under electrochemical conditions is within the experimental error range (Supplementary Tables 7–9).

### Calculation of the limit of detection

The limit of detection was calculated according to IUPAC specifications^53,54^:

5 $${\bar{x}}_{{\rm{B}}}=\mathop{\sum }\nolimits_{j=1}^{{n}_{{\rm{B}}}}{x}_{{\rm{B}}}/{n}_{{\rm{B}}}$$

6 $${{s}_{{\rm{B}}}}^{2}=\mathop{\sum }\nolimits_{j=1}^{{n}_{{\rm{B}}}}{({x}_{{\rm{B}}}\mbox{-}{\bar{x}}_{{\rm{B}}})}^{2}/({n}_{{\rm{B}}}-1)$$

7 $${x}_{{\rm{L}}}\,={\bar{x}}_{{\rm{B}}}+k\times {s}_{{\rm{B}}}$$

8 $${c}_{{\rm{L}}}=({x}_{{\rm{L}}}-{\bar{x}}_{{\rm{B}}})/m$$

where *x*_B_ is the blank measurement value, *n*_B_ is the number of replicates (20), ${\bar{x}}_{{\rm{B}}}$ is the mean value of the blank responses, *s*_B_ is the standard deviation, *k* is a numerical factor indicative of the confidence level obtained (3), *x*_L_ is the smallest discernible analytical signal, *m* is the analytical sensitivity, *c*_L_ is the limit of detection.

### Determination of NH_3_ and NO_*x*_ contamination

NH_3_ contamination was determined by the indophenol blue method. For ^14^N_2_, commercial gas or purified gas were continuously fed into 30 ml of deionized water at a flow rate of 30 sccm using a mass flow controller for 12 h. For ^15^N_2_, the deionized water was purged with commercial gas or purified gas for 30 min at a flow rate of 10 sccm using a mass flow controller before the experiments, and the gas was circulated for 12 h. The electrolyte (2 ml) was mixed with sodium hydroxide solution containing sodium citrate and salicylic acid (2 ml), followed by the addition of 0.05 M sodium hypochlorite (1 ml) and 1 wt% sodium nitroferricyanide (0.2 ml). After 3 h, the absorption spectrum was acquired using an ultraviolet-visible spectrophotometer. The concentration–absorbance curves were calibrated using standard ammonium sulfate solutions at a series of concentrations in the solvent. The weighted linear regression model was used for calibration.

NO or NO_2_ contamination was determined using the *N*-(-1-naphthyl)-ethylenediamine dihydrochloride spectrophotometric method. The chromogenic agent was obtained by dissolving sulfanilic acid (0.5 g) in deionized water (90 ml) and acetic acid (5 ml), followed by adding *N*-(1-naphthyl)-ethylenediamine dihydrochloride (5 mg) and bringing the solution to 100 ml. The chromogenic agent and deionized water were mixed with volume ratio of 1:4 to be used as the absorption liquid. Then commercial gas or purified gas was first passed through an oxidation tube and then fed into the absorption liquid for a period of time. For ^14^N_2_, commercial gas or purified gas were continuously fed into 30 ml absorption liquid at a flow rate of 30 sccm using a mass flow controller for 12 h. For ^15^N_2_, 30 ml absorption liquid was purged with commercial gas or purified gas for 30 min at a flow rate of 10 sccm using a mass flow controller, and the gas was circulated for 12 h. After standing in darkness for another 15 min, the absorption spectrum was measured using an ultraviolet-visible spectrophotometer. The concentration–absorbance curves were prepared using a standard sodium nitrite solution at a series of concentrations in the solvent. The weighted linear regression model was used for calibration.

N_2_O contamination was determined by performing the gas chromatography (Agilent 990, USA) experiments with commercial gas and purified gas^52^.

### In situ characterization

Different tailor-made cells were customized for different in situ characterizations. For all in situ characterization techniques, a graphite rod was used as the counter electrode, and Ag/AgCl/saturated KCl was used as the reference electrode. N_2_- or Ar-saturated LiCl solutions with different concentrations were used as the electrolyte. The electrolyte was continuously bubbled with N_2_ at a flow rate of 30 sccm using a mass flow controller. For in situ FTIR measurements, a glassy carbon electrode coated with the catalyst was used as the working electrode. The in situ FTIR spectra were recorded by FTIR spectroscopy (NEXUS-870, Nicolet Instrument Co., USA). For in situ Raman and in situ XRD measurements, carbon paper coated with the catalyst was used as the working electrode. In situ XRD spectra were acquired using a Bruker D8 Advance system (Germany). In situ Raman spectra were recorded on a Horiba Jobin Yvon HR evolution instrument (France).

### Electrochemical NRR measurements

The electrochemical measurements were conducted in a gas-tight H-type cell separated by the Celgard membrane with hydrophilic treatment. Both the cathode chamber and anode chamber contained 30 ml of LiCl electrolyte. A graphite rod was used as the counter electrode, and Ag/AgCl/saturated KCl was used as the reference electrode. Before the measurement, the electrolyte was purged with purified nitrogen for 30 min. During the electrochemical NRR measurements, purified N_2_ was continuously fed into the cathodic compartment with a properly positioned sparger to ensure that the whole cathode was hit by N_2_ gas bubbles. The electrolyte was continuously bubbled with N_2_ at a flow rate of 30 sccm using a mass flow controller.

For NRR experiments, the potentiostatic tests were conducted in LiCl aqueous solutions at different potentials, including −0.1, −0.2, −0.3, −0.4, and −0.5 V vs. RHE. An in-line acid trap of the cathode chamber filled with 0.1 M HCl was set to avoid volatilization of the produced ammonia. The electrolytes in the cathode and acid trap were both collected and analysed for quantitative measurements. The total ammonia yield was the summation of that in LiCl and in 0.1 M HCl.

### Determination of ammonia from the NRR process

For the colorimetric method, the concentration of ammonia in the LiCl solution was determined using the same method as that in distilled water, except that the standing time was changed to 10 min. The concentration of ammonia in the HCl solution was determined using the same method as that in distilled water. For the NMR measurements, the standard curve was constructed by measuring a series of areas of the NMR peak for the reference solutions at different ammonium sulfate concentrations. The weighted linear regression model was used for calibration. The electrolytes in the cathode and acid trap were mixed and concentrated to 30 ml after electrolysis. Then, 0.5 ml of the resulting liquid was taken out, followed by adding 0.1 ml of DMSO-d6. Maleic acid was used as the internal standard. The produced ammonia was quantified by using ^1^H NMR spectroscopy (Agilent 600 MHz).

### Determination of hydrazine

The hydrazine present in the LiCl solution was determined using the Watt and Chrisp method. The chromogenic agent was obtained by mixing para-(dimethylamino) benzaldehyde (5.99 g), HCl (30 ml), and ethanol (300 ml). Typically, the chromogenic agent (5 ml) was added into the electrolyte (5 ml) and stirred for 10 min at room temperature. The absorption spectrum was acquired using an ultraviolet-visible spectrophotometer. The concentration–absorbance curves were calibrated using a standard hydrazine monohydrate solution at a series of concentrations. The weighted linear regression model was used for calibration. The concentration of produced hydrazine in the 0.1 M HCl solution was determined using the same method.

### Determination of hydrogen

Gas chromatography (Agilent 7890B, USA) was used to perform the hydrogen evolution analysis.

### Yield rate and Faradaic efficiency of ammonia and hydrogen

The ammonia yield rate and Faradaic efficiency were calculated as follows:

9 $${\rm{FE}}({{\rm{NH}}}_{3})=[3F\times c({{\rm{NH}}}_{3})\times V]/Q$$

10 $${\rm{Yield}}\,{\rm{rate}}({{\rm{NH}}}_{3})=[17c({{\rm{NH}}}_{3})\times V]/(t\times A)$$

where *Q* is the total charge passed through the electrode, *V* is the volume of the electrolyte, *c*(NH_3_) is the measured ammonia concentration, *F* is the Faraday constant (96,485 C mol^−1^), *A* is the surface area of the working electrode, and *t* is the electrolysis time (3600 s).

The Faradaic efficiency of the hydrogen evolution was calculated as follows:

11 $${\rm{FE}}({{\rm{H}}}_{2})=2F\times v({{\rm{H}}}_{2})\times G\times {p}_{0}/R\times {T}_{0}\times {i}_{{\rm{total}}}$$

where *v*(H_2_) is the volume concentration of hydrogen in the exhaust gas from the electrochemical cell, *G* is the gas flow rate, *p*_0_ = 1.01 × 10^5^ Pa, *R* = 8.314 J mol^−1^ K^−1^, and *i*_total_ is the steady-state cell current.

The yield rate of hydrogen evolution was calculated as follows:

12 $${\rm{Yield}}\,{\rm{rate}}({{\rm{H}}}_{2})=[Q\times {\rm{FE}}({{\rm{H}}}_{2})\times {M}_{{\rm{r}}}({{\rm{H}}}_{2})]/(e\times {N}_{{\rm{A}}}\times 2)\times (t\times A)$$

where *M*_r_(H_2_) is the relative molecular mass of H_2_, *e* is the electron charge (1.602 × 10^−19^ C), and *N*_A_ is Avogadro’s constant (6.02 × 10^23^ mol^−1^).

### ^15^N_2_ isotopic labelling experiment

The labelling experiments were performed in a dedicated Argon glovebox with a home-made gas recirculation system to eliminate ^14^N_2_ and reuse expensive ^15^N_2_^37,55^. All the electrochemical cell components, including the purification traps, electrodes, electrolytes, and the absorption cell, were prepared and assembled in^.^ the Argon glovebox. The electrolytes used in the labelling experiments were also intensively degassed using a certain number of freeze–thaw cycles prior to its further storage and handling in the Argon glovebox. Before the labelling experiments, the purified ^15^N_2_ was purged for 30 min (approximately 300 ml) to displace the argon inside the system. Following this step, the system was switched to circulating mode and the labelling experiments were then performed at a ^15^N_2_ flow rate of 30 sccm using a mass flow controller. After the electrolytic reaction, the obtained ^15^NH_4_^+^-containing electrolyte was detected by ^1^H NMR. (^15^NH_4_)_2_SO_4_ and (^14^NH_4_)_2_SO_4_ were used as the benchmarks. The produced ^15^NH_3_ was quantified using the same method as that for ^14^NH_3_ either by the colorimetric method or NMR spectroscopy.

## Supplementary information

Supplementary Information

Description of Additional Supplementary Files

Supplementary Data 1

Supplementary Data 2

## Data Availability

The data that support the findings of this study are available within the article and its Supplementary Information files or from the corresponding author upon reasonable request. Source data are provided with this paper.
