# Supplementary material for: Salting-out effect promoting highly efficient ambient ammonia synthesis
Source: Nat Commun. 2021 May 27;12:3198. doi: 10.1038/s41467-021-23360-0 (PMC8160333; doi:10.1038/s41467-021-23360-0)
Supplement: Supplementary file 1 — Supplementary Information [file 41467_2021_23360_MOESM1_ESM.pdf]

# Supplementary Information

# Salting-out effect promoting highly efficient ambient ammonia synthesis

**Wang et al.**

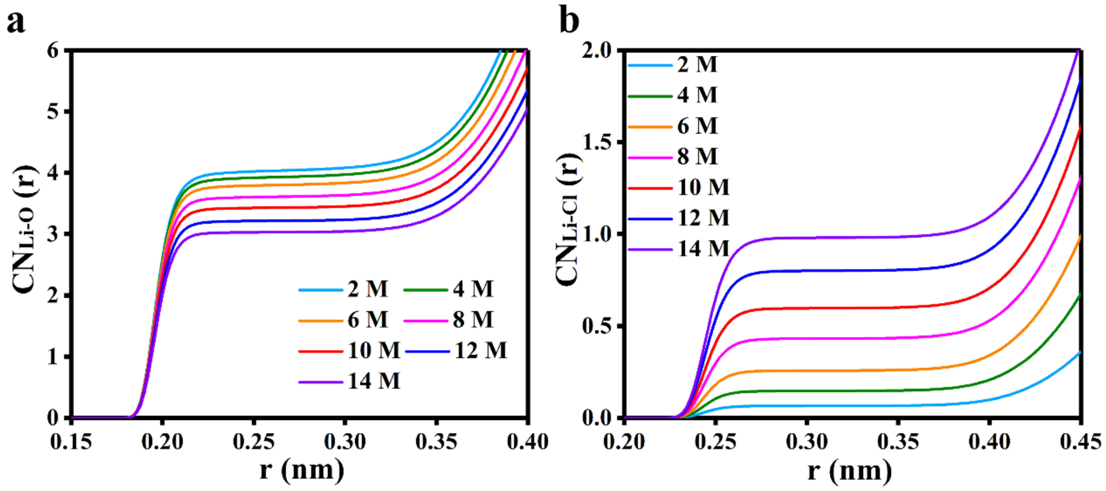


**Supplementary Figure 1.** Coordination number for the **a** $Li^+$-O and **b** $Li^+$-$Cl^-$ interactions.

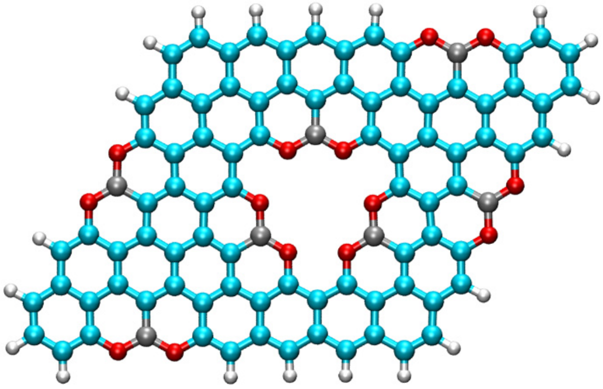

**Supplementary Figure 2.** The proposed configuration of the catalyst model for theoretical simulations. Silver, cyan, red, and white spheres represent B, C, O, and H atoms, respectively. Since the aim of theoretical simulations is to guide the experiments, the design of the catalyst model also took the actual situation into account. Therefore, vacancy and edges doped with boron and oxygen were considered in the catalyst model. Moreover, the initial configuration was energy-minimized and equilibrated to obtain the most stable state before further simulations.

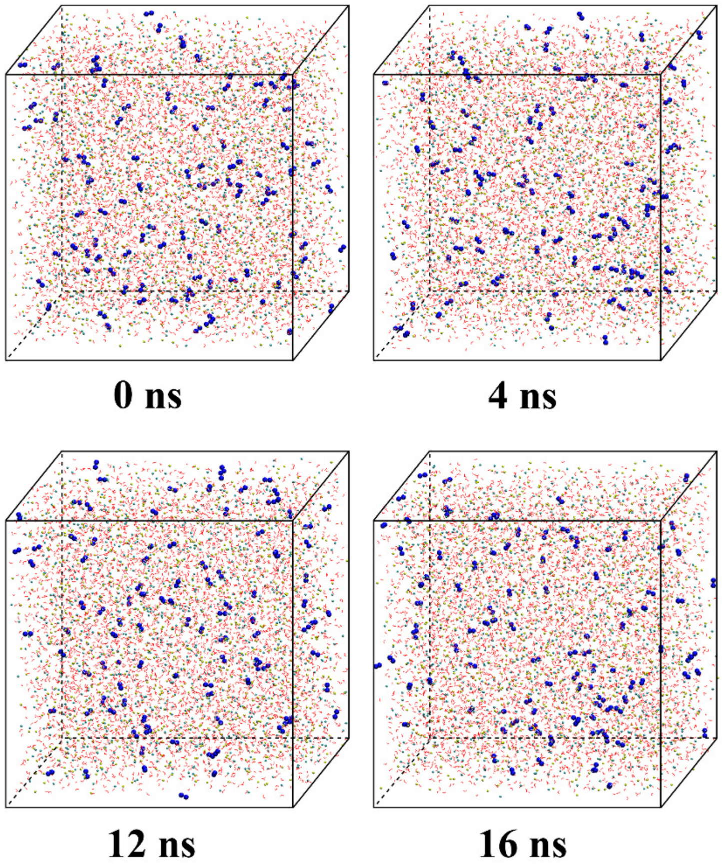


**Supplementary Figure 3.** Snapshots of the heterogeneous catalysis system with the absence of the catalyst and 10 M LiCl electrolyte as a function of time.

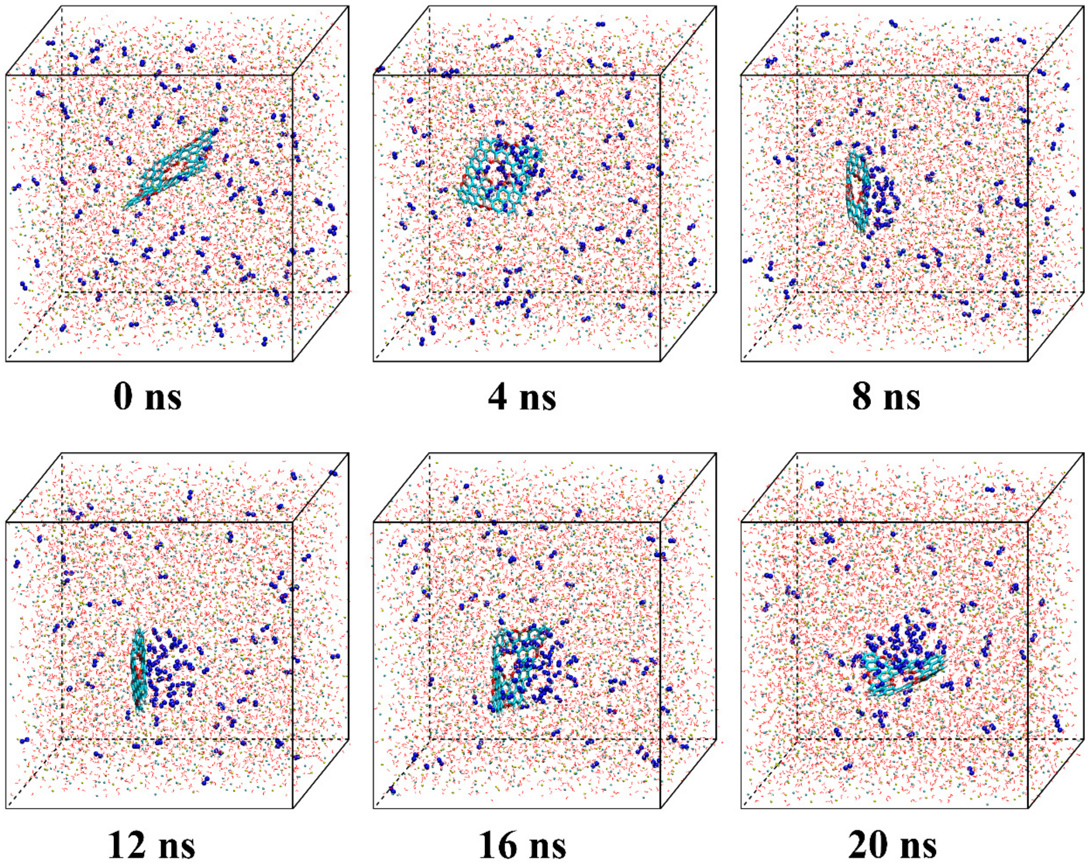


**Supplementary Figure 4.** Snapshots of the heterogeneous catalysis system with the presence of the catalyst and 10 M LiCl electrolyte as a function of time.

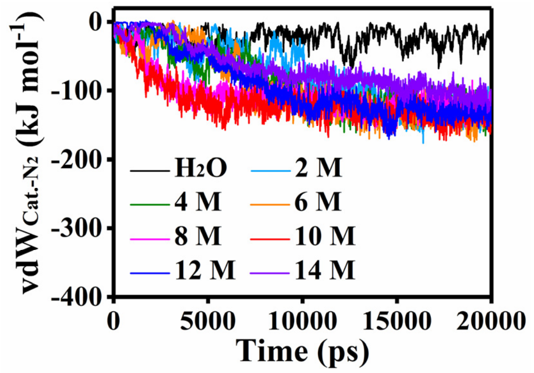


**Supplementary Figure 5.** Van der Waals interactions between the catalyst and $N_2$ molecules.

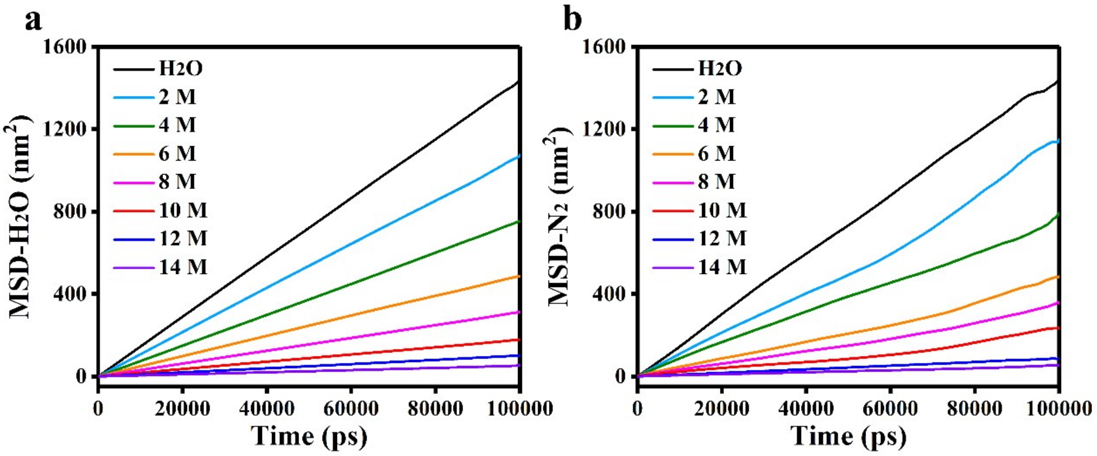


**Supplementary Figure 6.** Mean square displacement for **a** $H_2O$ and **b** $N_2$.

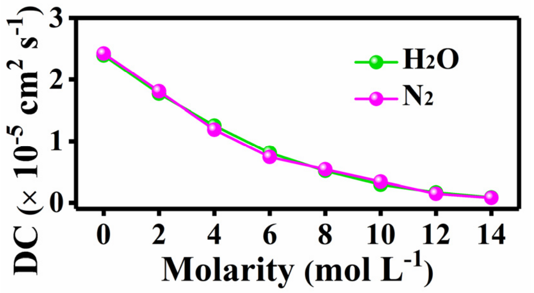


**Supplementary Figure 7.** Diffusion coefficients (DC) of $H_2O$ and $N_2$ as a function of salt concentration.

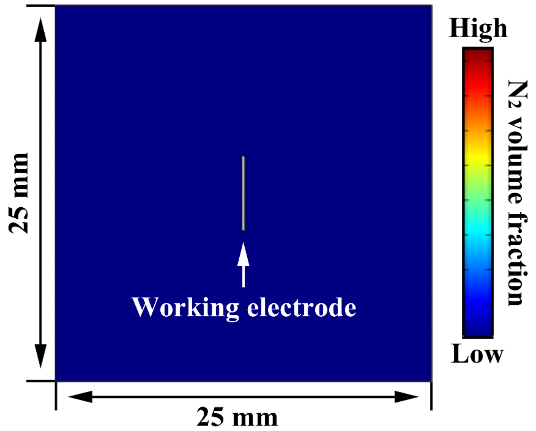


**Supplementary Figure 8.** The modeling of the heterogeneous catalysis system in finite element simulations. The white rectangle represents the working electrode (WE).

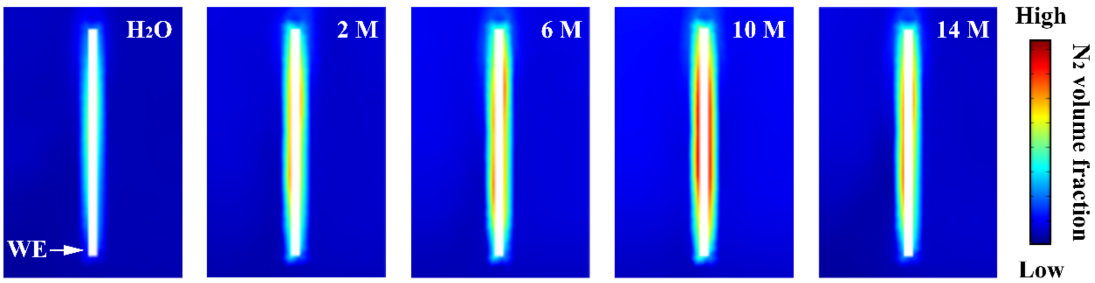


**Supplementary Figure 9.** Nitrogen distribution profiles at the WE surface in different electrolytes after 100 s in finite element simulations.

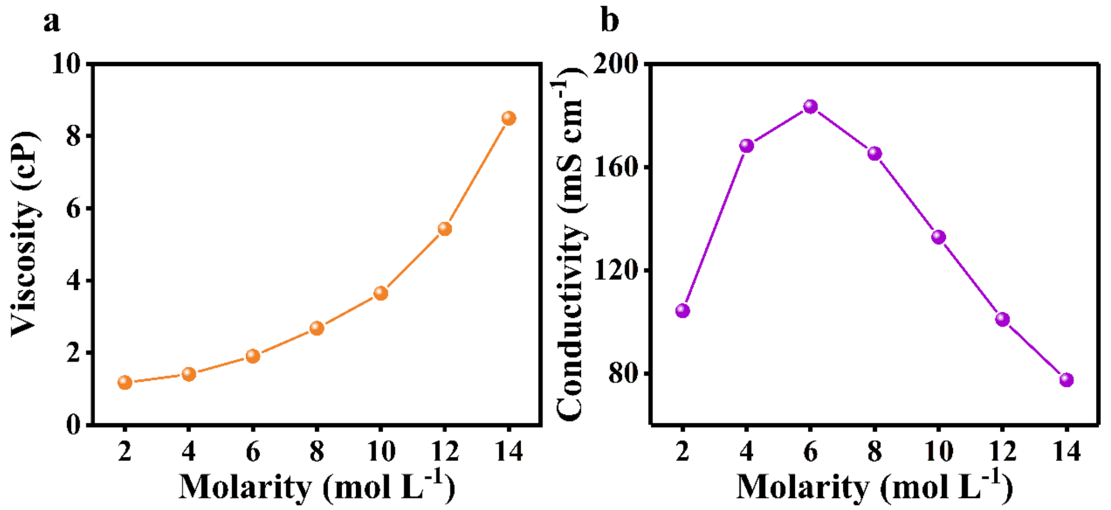


**Supplementary Figure 10. a** Viscosity and **b** conductivity of the LiCl solutions with different concentrations.

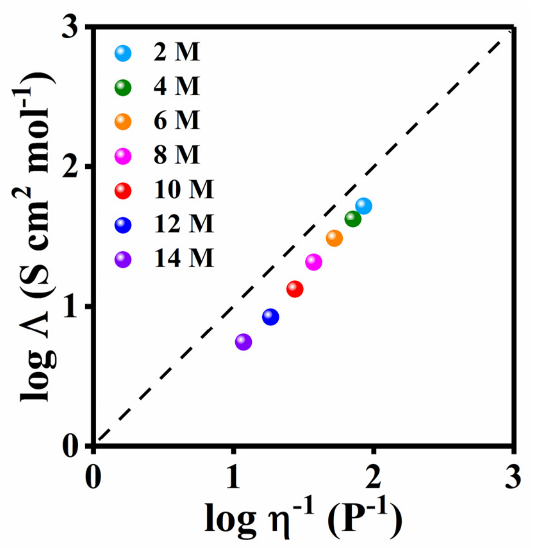


**Supplementary Figure 11.** Walden plot of the LiCl solutions with different concentrations. The ideal line position is based on the properties of dilute aqueous KCl solution.

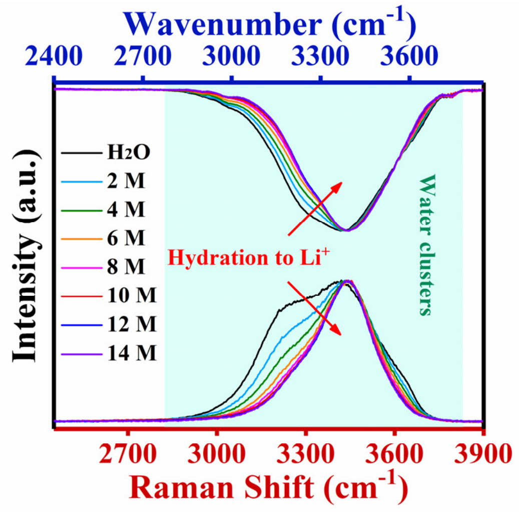


**Supplementary Figure 12.** Raman and FTIR spectra of the LiCl solutions with different concentrations.

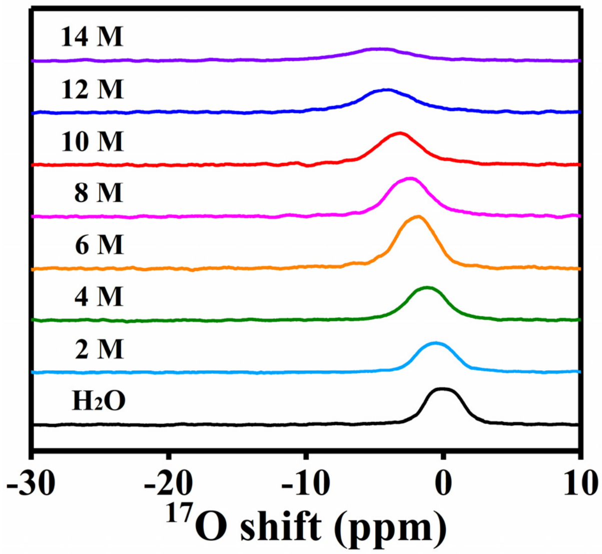


**Supplementary Figure 13.** $^{17}O$ NMR spectra of the LiCl solutions with different concentrations.

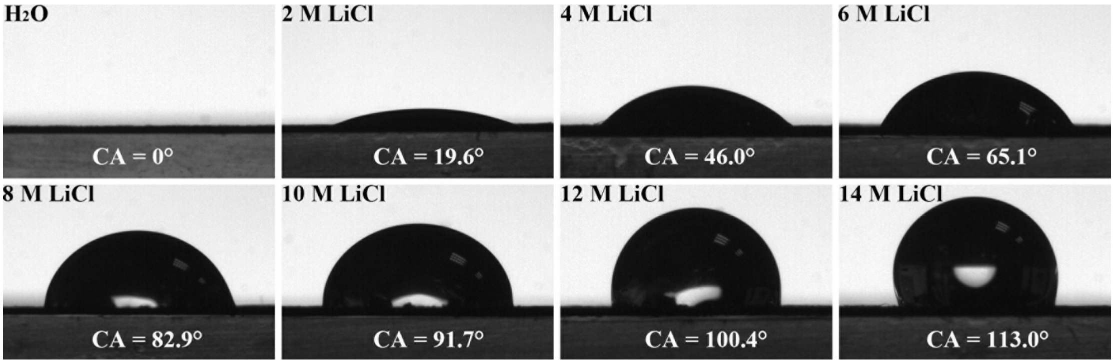


**Supplementary Figure 14.** The contact angle between the substrate and the solution droplet of the LiCl solutions with different concentrations.

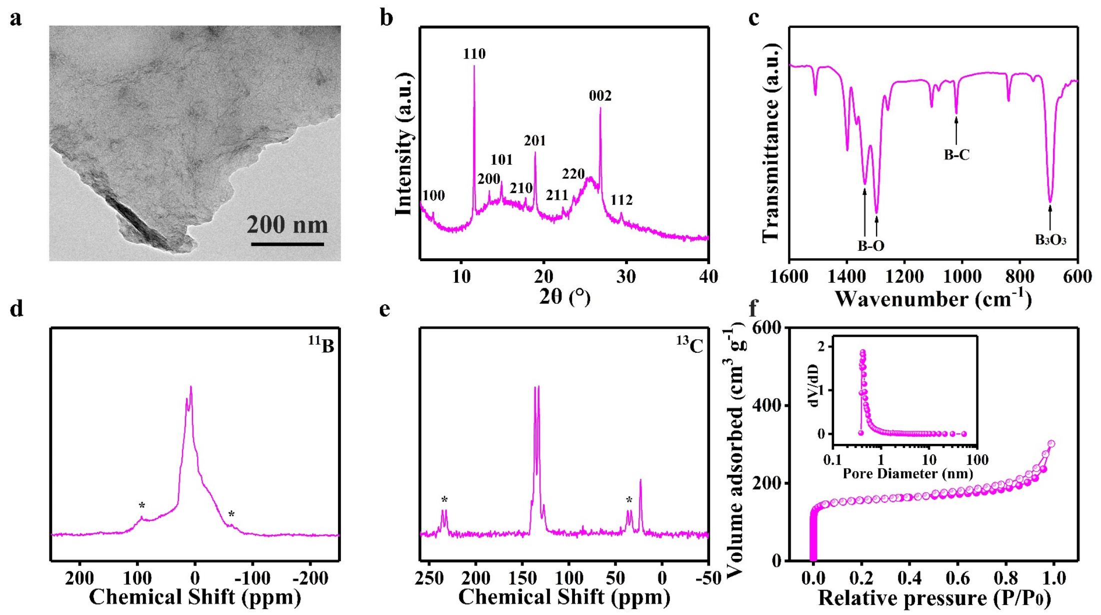


**Supplementary Figure 15. Physical characterization of B-COF/GO. a** TEM image, **b** XRD pattern, and **c** FTIR spectrum of B-COF. **d** $^{11}$B and **e** $^{13}$C CP/MAS NMR spectra of B-COF. **f** BET surface area and pore size distribution (inset) of B-COF. COF-1 are uniformly coated on the GO substrate as suggested by the TEM image. The XRD pattern, FTIR spectrum, $^{11}$B, and $^{13}$C CP/MAS NMR spectra all show the characteristic peaks of COF-1. B-COF shows a BET surface area of 482.75 m$^2$ g$^{-1}$, with a large fraction of pore volume originated from micropores.

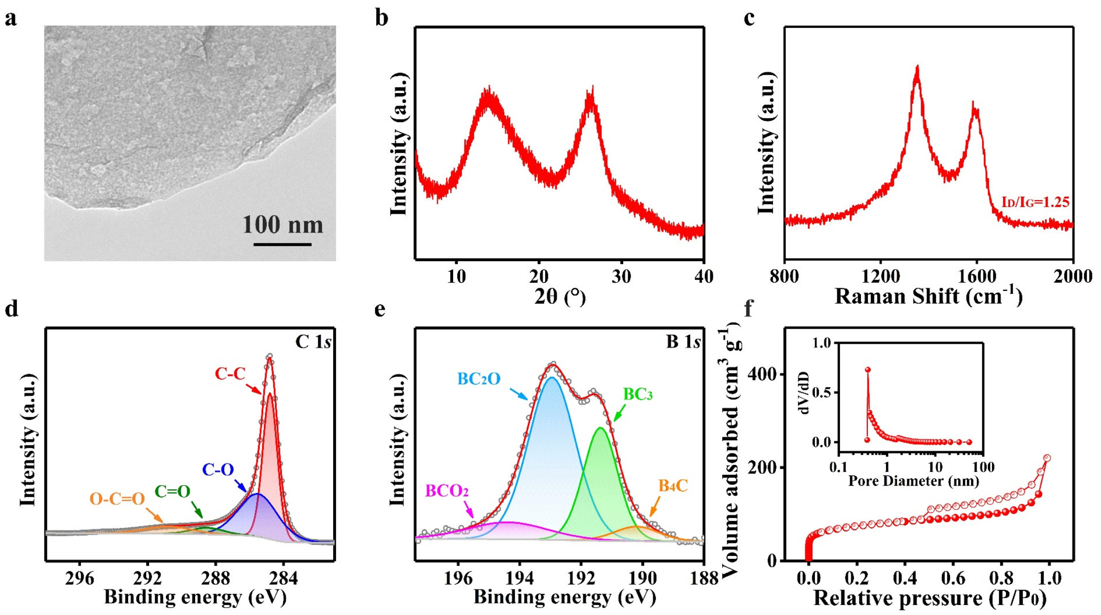


**Supplementary Figure 16. Physical characterization of SAB/C. a** TEM image, **b** XRD pattern, and **c** Raman spectrum of SAB/C. **d** High resolution C 1*s* and B 1*s* spectra of SAB/C. **f** BET surface area and pore size distribution (inset) of SAB/C. The SAB/C inherited the geometry of precursor B-COF, forming porous carbon nanosheets. Its transformation into amorphous carbon is confirmed by the XRD and Raman pattern. The high-resolution C 1*s* spectrum is consisted of four characteristic peaks, corresponding to the C–C, C–O, C=O, and O–C=O structures, respectively. The high-resolution B 1*s* peak is fitted into four peaks, assigned to structures of $B_4C$, $BC_3$, $BC_2O$, and $BCO_2$. SAB/C shows a BET surface area of 243.10 m$^2$ g$^{-1}$. The decreased BET surface area compared with B-COF may be due to the collapse of the COF structure.

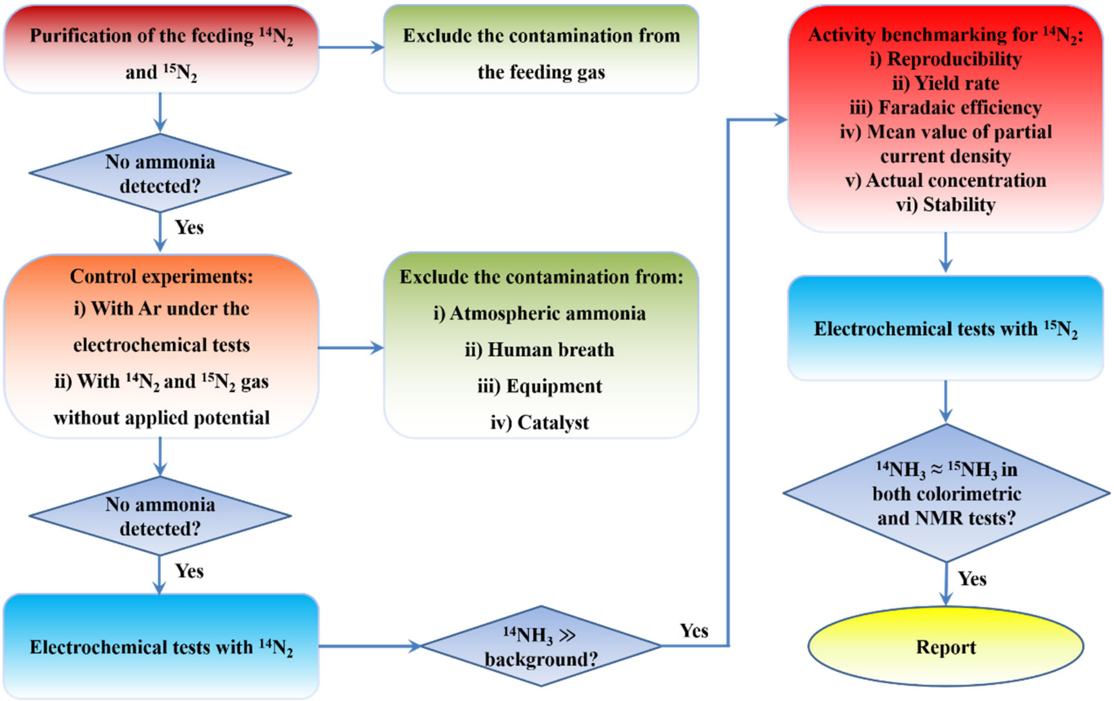


**Supplementary Figure 17.** Rigorous experimental protocol for reliable proof of the occurrence of the electrochemical nitrogen reduction reaction.

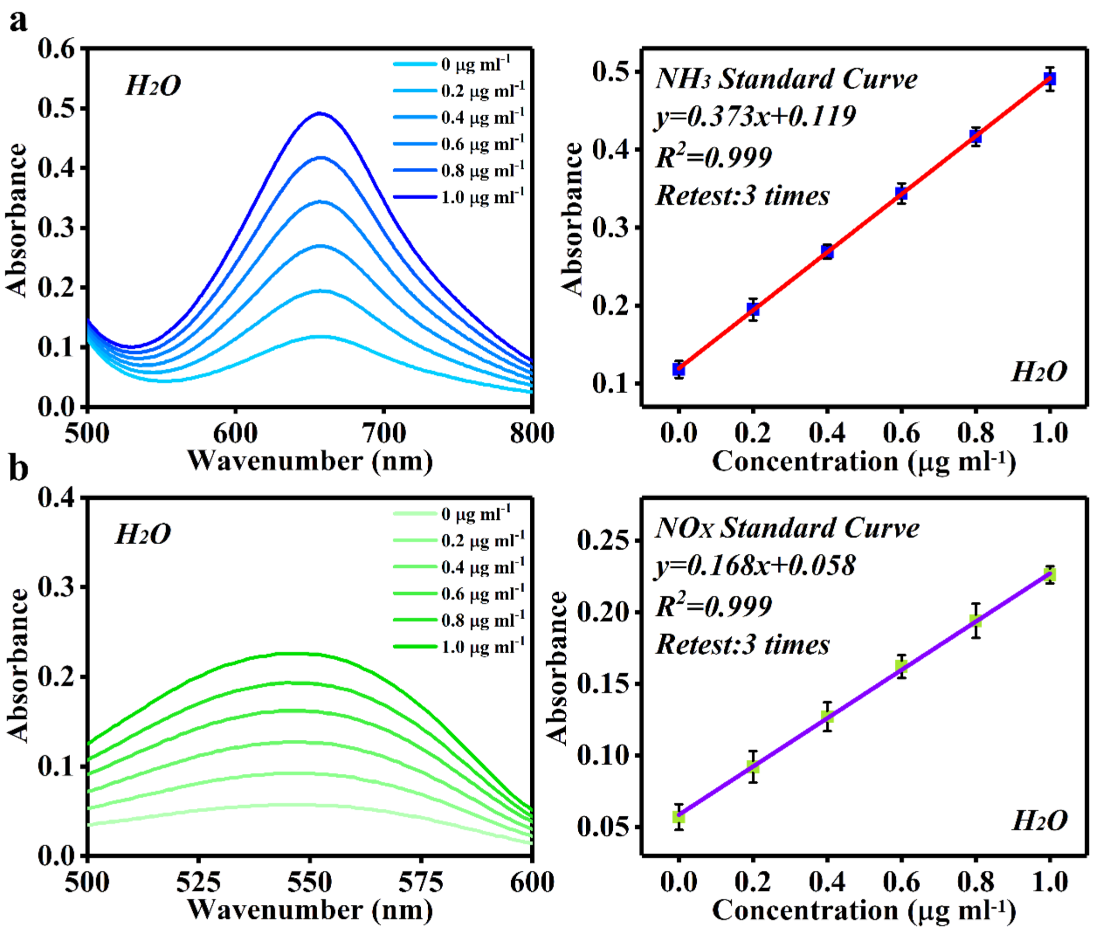


**Supplementary Figure 18.** Calibration curves for **a** the colorimetric $^{14}NH_3$ assay using the indophenol blue method and **b** the colorimetric $NO_x$ assay using the N-(-1-naphthyl)-ethylenediamine dihydrochloride spectrophotometric method in deionized water. The error bars correspond to the standard deviations of measurements over three separately prepared samples under the same conditions.

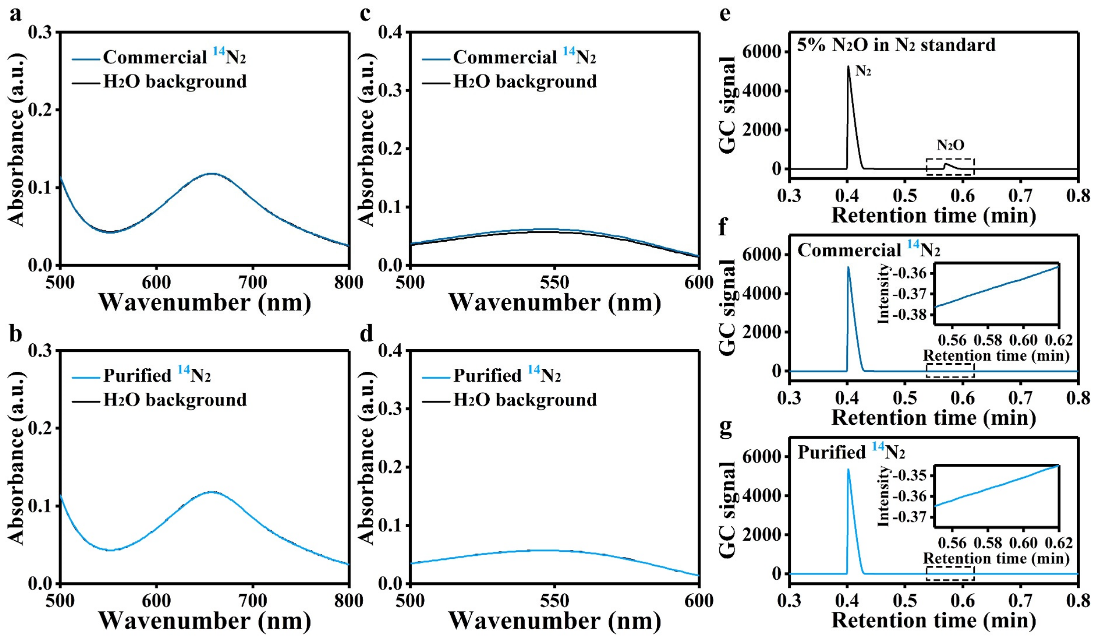


**Supplementary Figure 19. a-b** The UV-vis absorption spectra of the deionized water treated by commercial $^{14}N_2$ and purified $^{14}N_2$ using indophenol blue method. **c-d** The UV-vis absorption spectra of the absorption liquid using N-(-1-naphthyl)-ethylenediamine dihydrochloride spectrophotometric method. **e-g** Gas chromatography (GC) spectra of 5% $N_2O$ in $N_2$ standard, commercial $^{14}N_2$, and purified $^{14}N_2$.

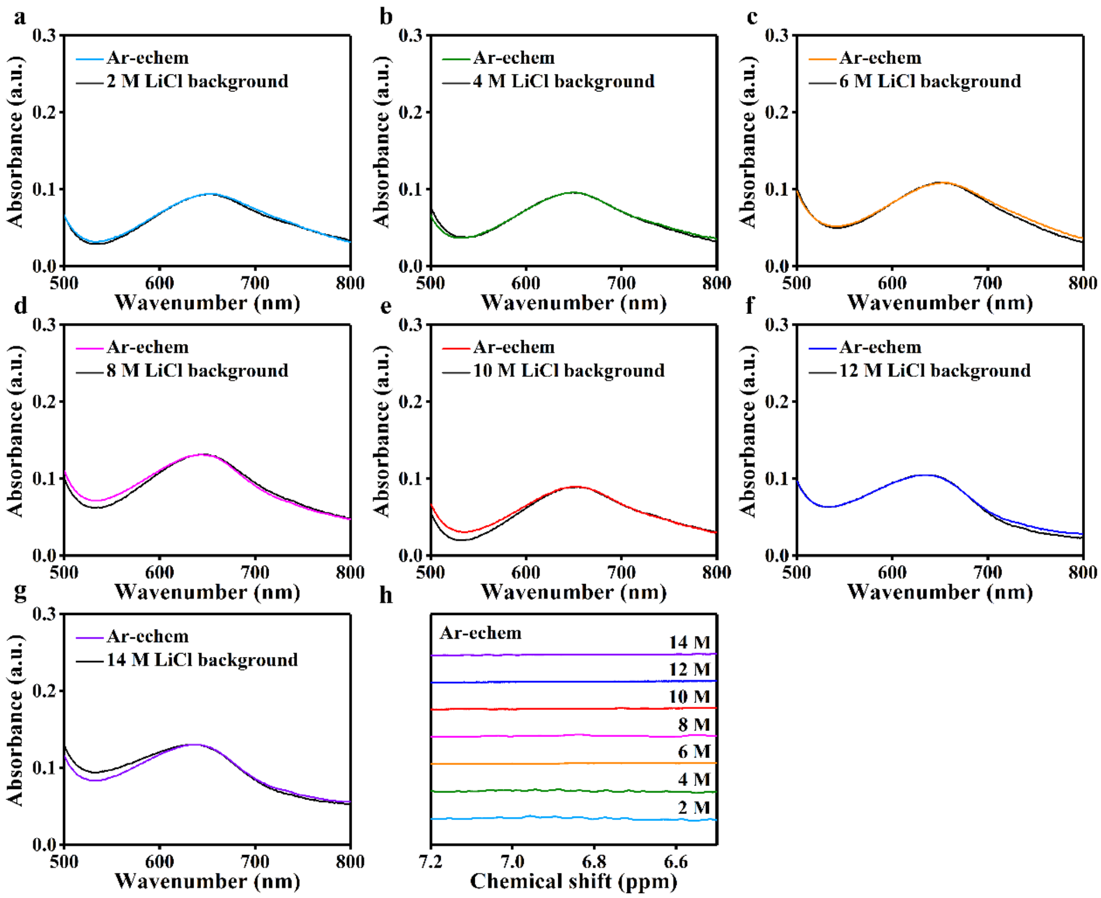


**Supplementary Figure 20. a-g** The UV-vis absorption spectra and **h** $^1$H NMR spectra of the electrolyte saturated with Ar under the electrochemical tests. Both colorimetric method and NMR method confirm that ammonia contamination is below the limit of detection in control experiments.

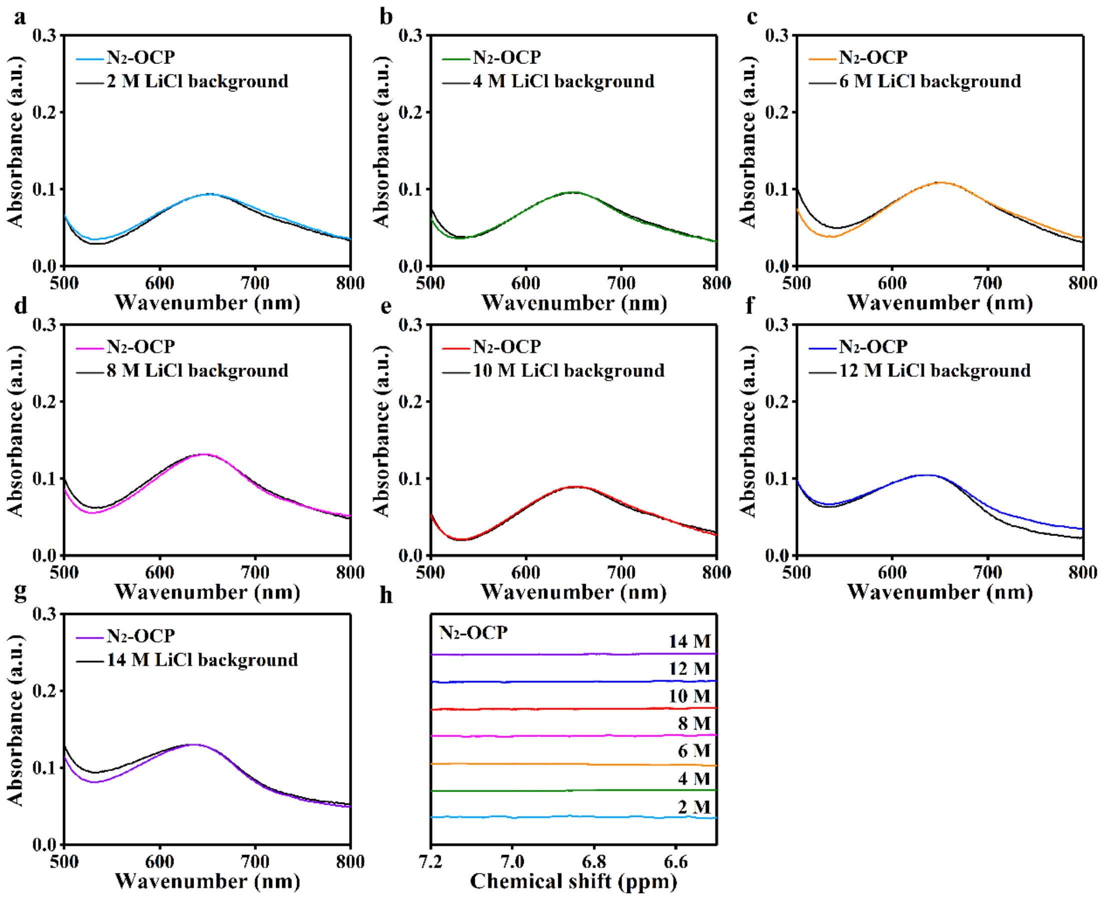


**Supplementary Figure 21. a-g** The UV-vis absorption spectra and **h** $^{1}$H NMR spectra of the electrolyte saturated with $N_2$ at open circuit potential (OCP). Both colorimetric method and NMR method confirm that ammonia contamination is below the limit of detection in control experiments.

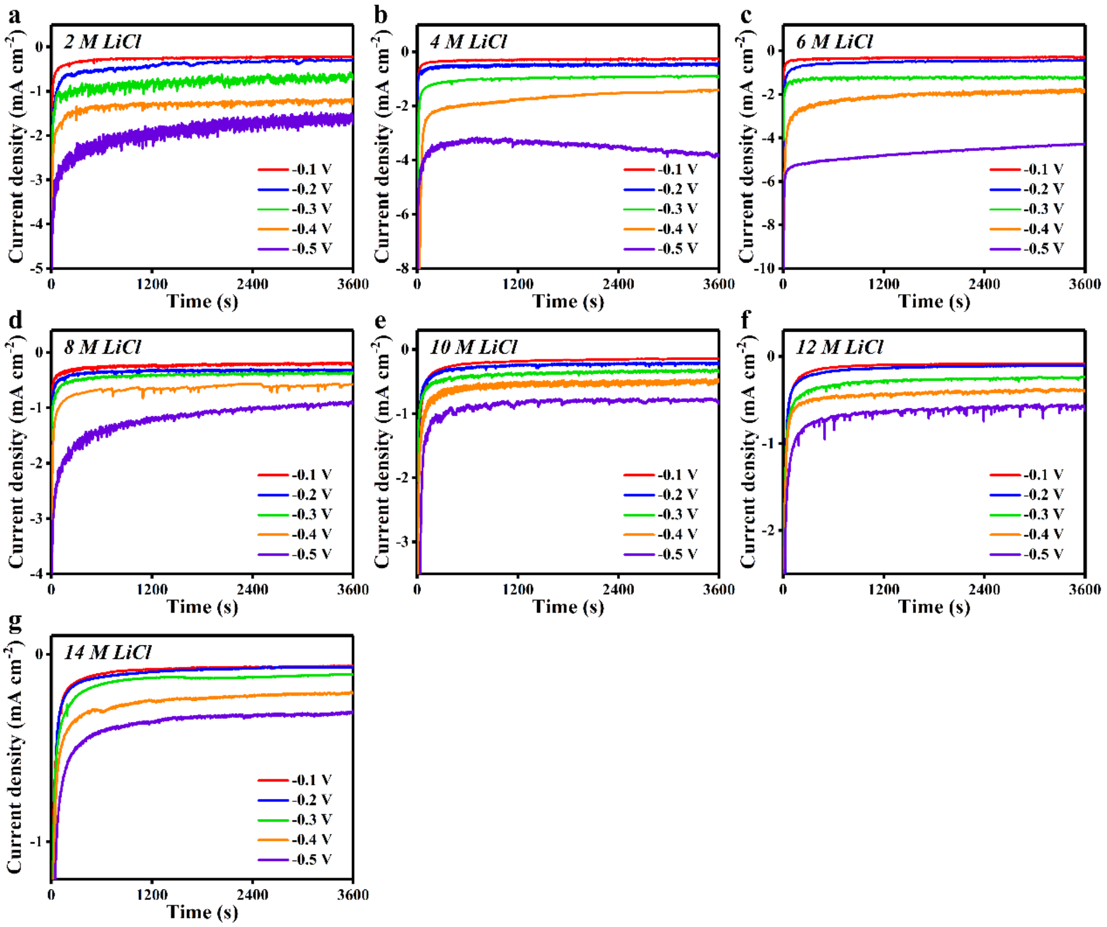


**Supplementary Figure 22.** Chronoamperometry results of different systems at the corresponding potentials.

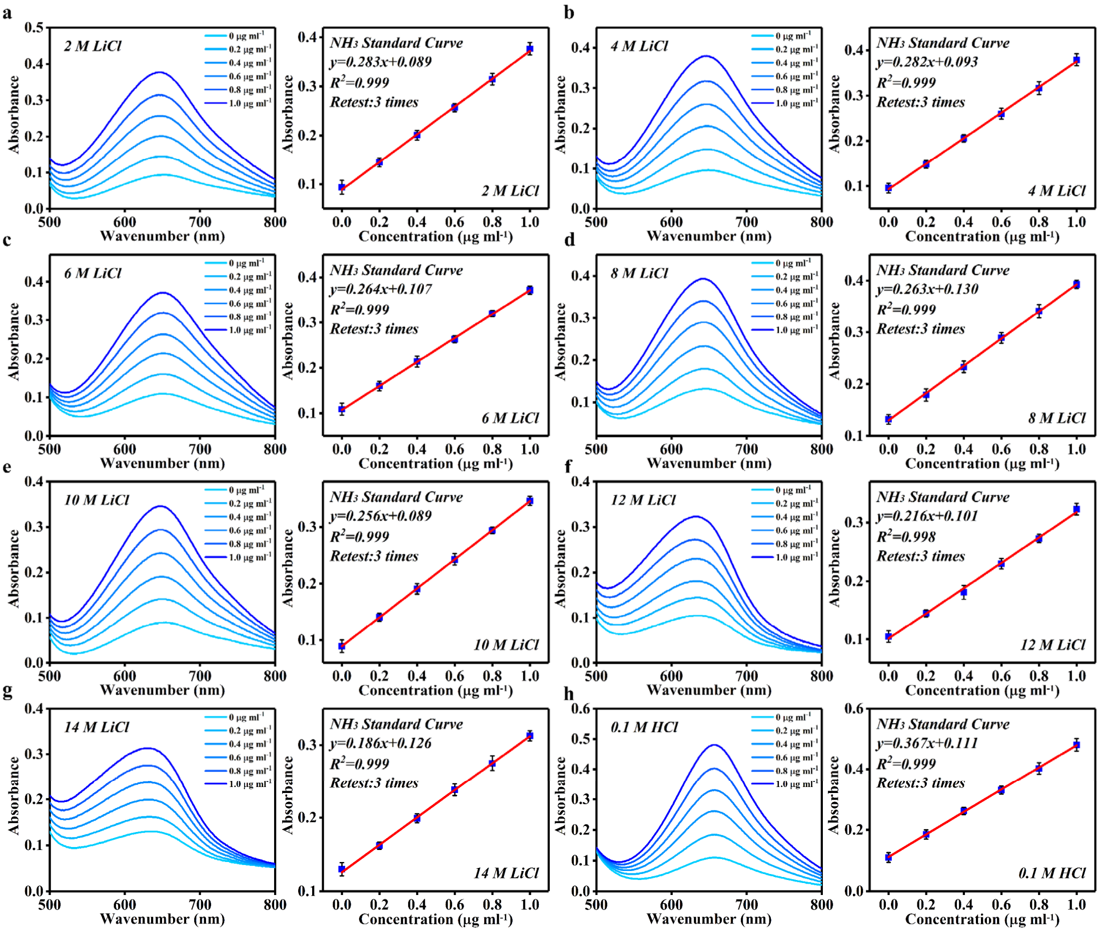


**Supplementary Figure 23.** Calibration curves for the colorimetric $^{14}NH_3$ assay using the indophenol blue method in different electrolytes. The error bars correspond to the standard deviations of measurements over three separately prepared samples under the same conditions.

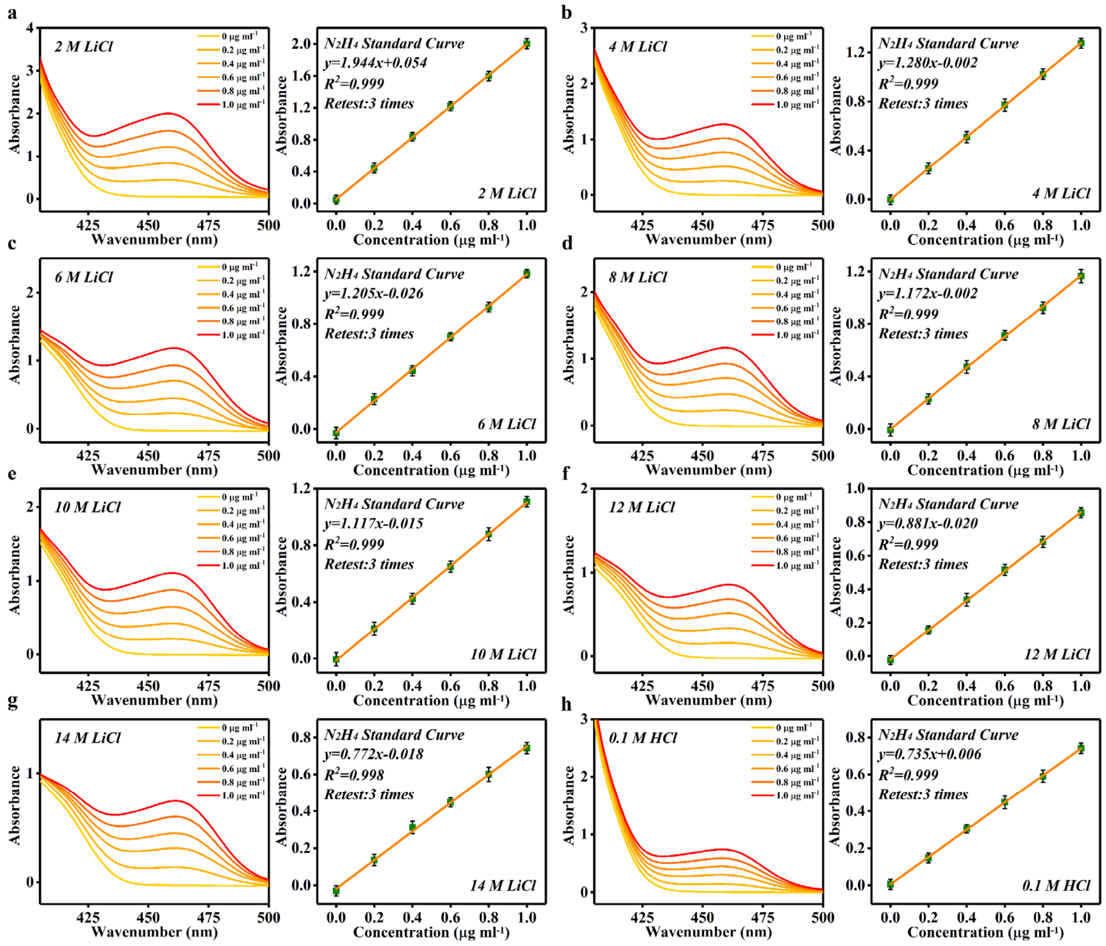


**Supplementary Figure 24.** Calibration curves for the colorimetric $N_2H_4$ assay using the Watt and Chrisp method in different electrolytes. The error bars correspond to the standard deviations of measurements over three separately prepared samples under the same conditions.

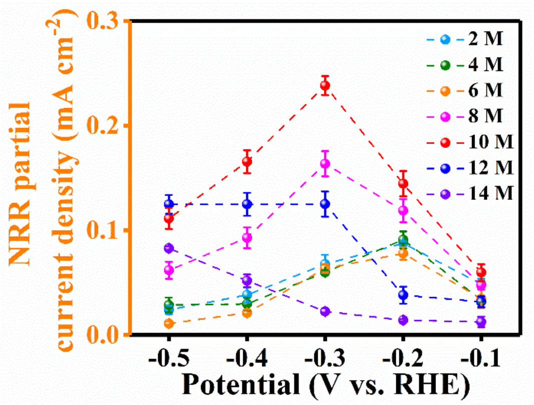


**Supplementary Figure 25.** NRR partial current density in different systems at various potentials. The error bars correspond to the standard deviations of measurements over three separately prepared samples under the same conditions.

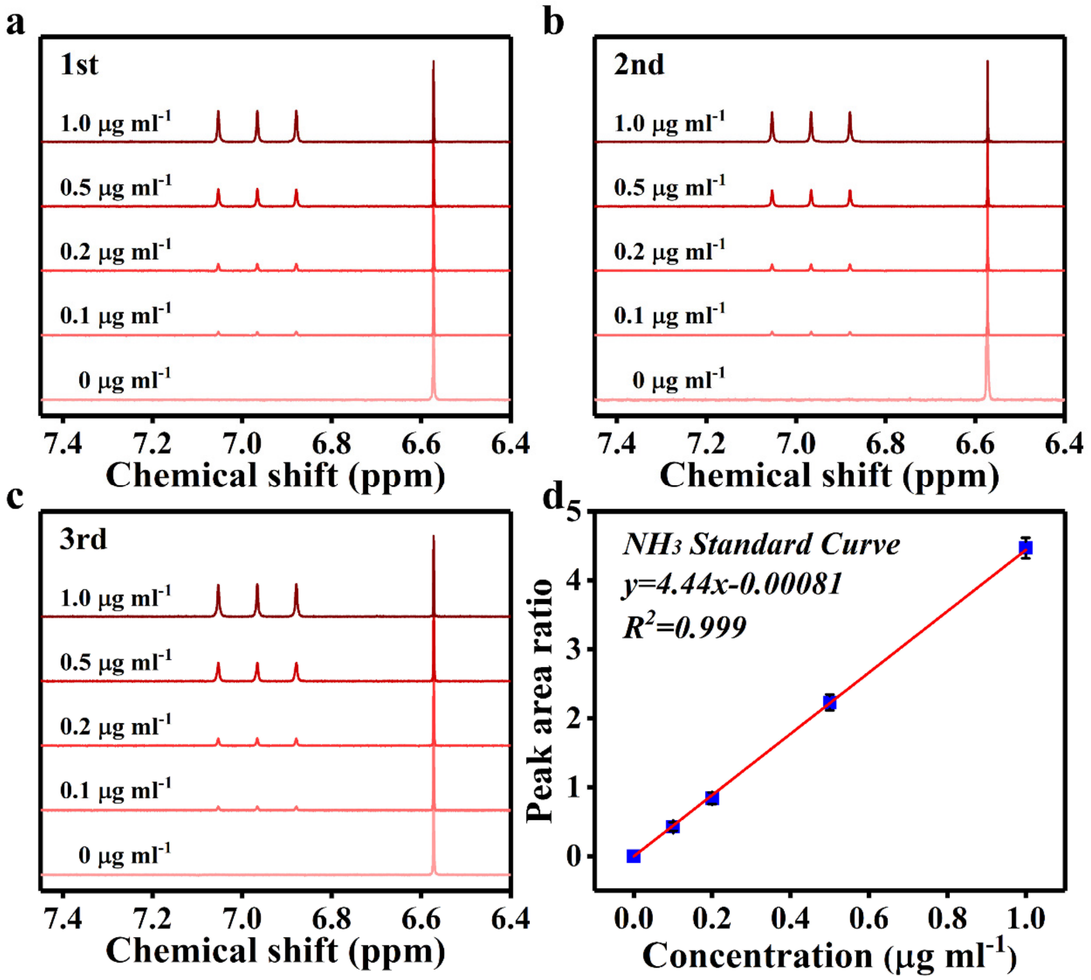


**Supplementary Figure 26. a-c** $^1$H NMR spectra of $^{14}NH_4^+$ with different normal concentrations from all three independent tests and **d** the corresponding standard curve for $^{14}NH_3$ concentration *vs.* peak area ratio. The error bars correspond to the standard deviations of measurements over three separately prepared samples under the same conditions. The calibration plots for the data points with error bars are almost perfectly linear, with an $R^2$ value of 0.999.

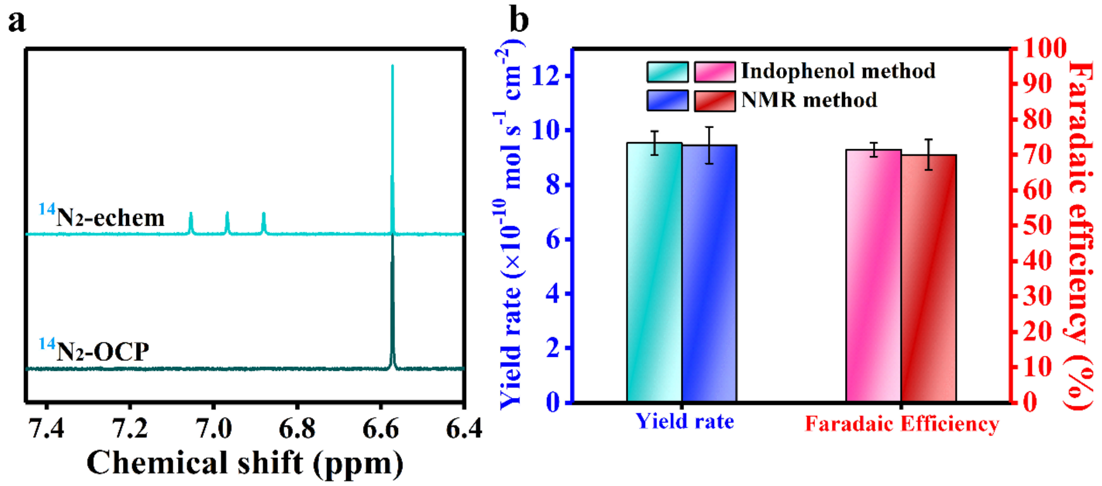


**Supplementary Figure 27. a** NMR spectra of 10 M LiCl electrolyte after electrolysis with $^{14}N_2$ as the feeding gas at OCP and –0.3 V vs. RHE, respectively. **b** Comparison of $NH_3$ yield rate and Faradaic efficiency at −0.3 V vs. RHE either by indophenol or NMR method. The error bars correspond to the standard deviations of measurements over three separately prepared samples under the same conditions.

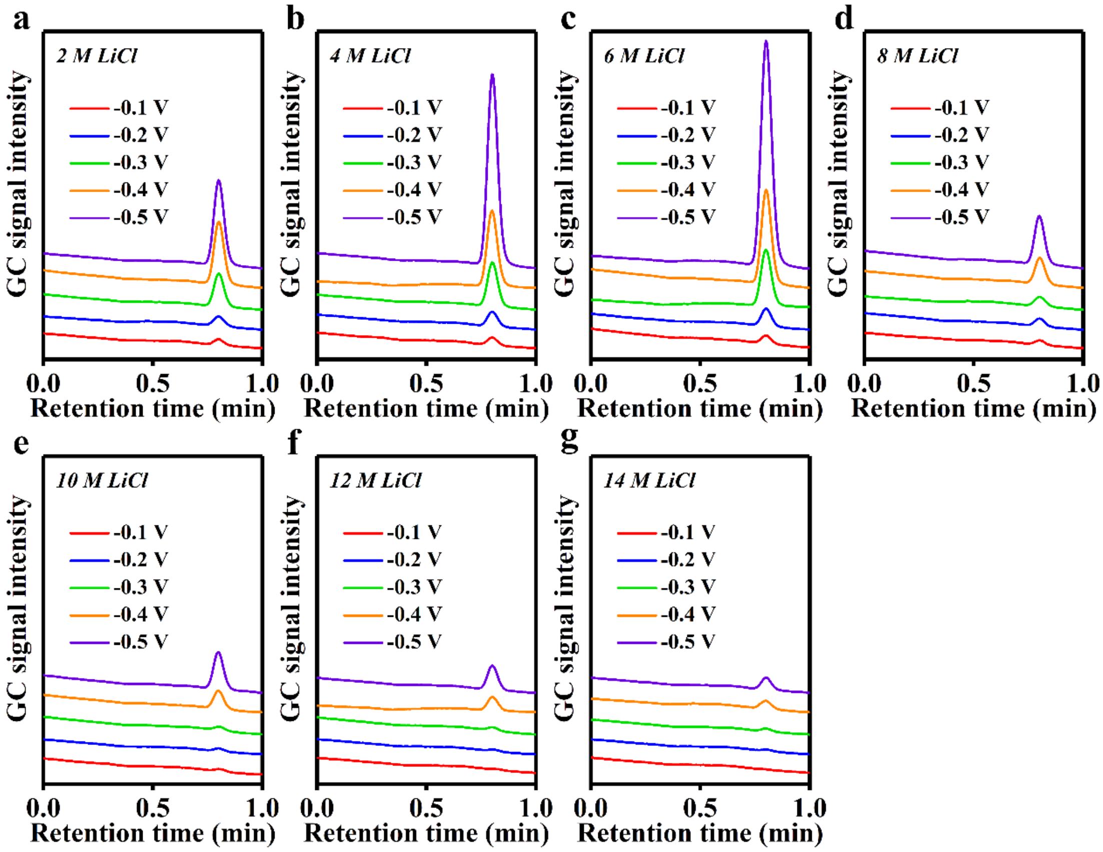


**Supplementary Figure 28.** GC spectra of the gas from the headspace of the cell with different electrolytes at various potentials.

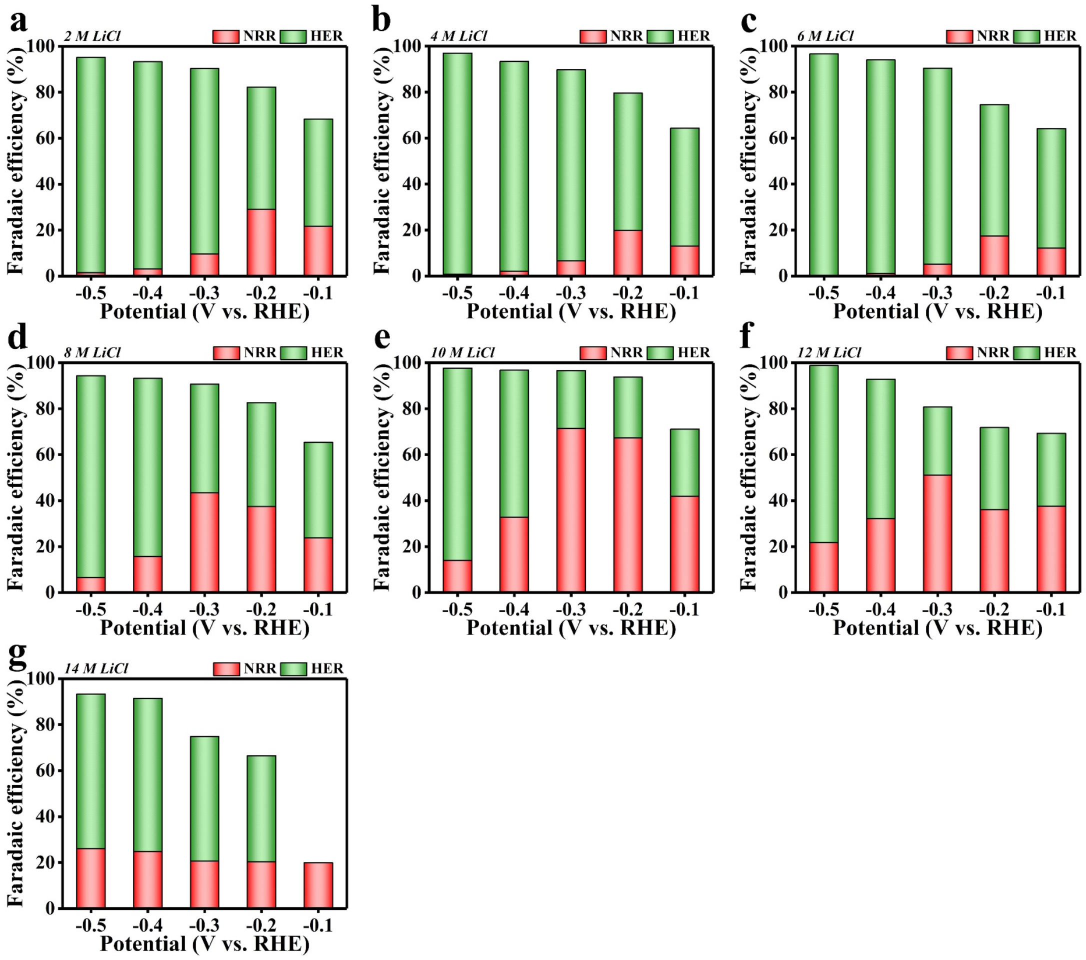


**Supplementary Figure 29.** The Faradaic efficiencies of NRR and HER in different electrolytes at various potentials. The unaccounted part may be attributed to the capacitance and adsorption behavior.

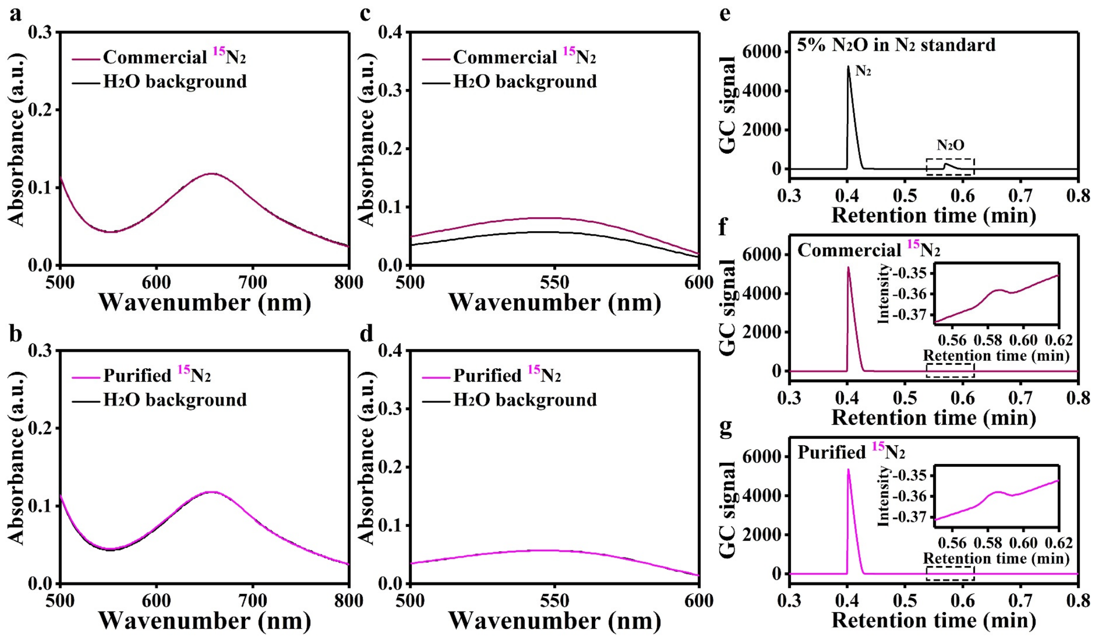


**Supplementary Figure 30. a-b** The UV-vis absorption spectra of the deionized water treated by commercial $^{14}N_2$ and purified $^{14}N_2$ using indophenol blue method. **c-d** The UV-vis absorption spectra of the absorption liquid using N-(-1-naphthyl)-ethylenediamine dihydrochloride spectrophotometric method. **e-g** GC spectra of 5% $N_2O$ in $N_2$ standard, commercial $^{15}N_2$, and purified $^{15}N_2$. Although commercial $^{15}N_2$ contains trace of $N_2O$ and it is hard to be excluded by the purification process, its influence to the ammonia yield rate is proved to be within the experimental error range.

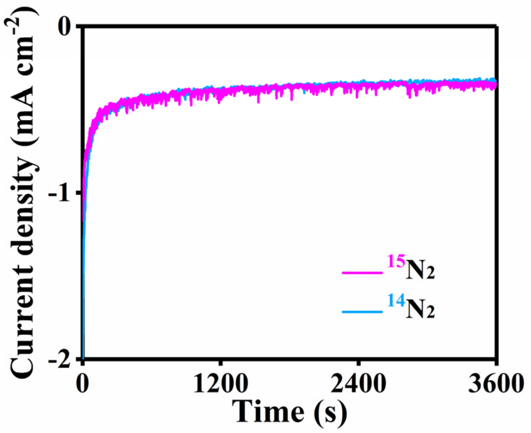

**Supplementary Figure 31.** Chronoamperometry results at –0.3 V vs. RHE with 10 M LiCl as the electrolyte.

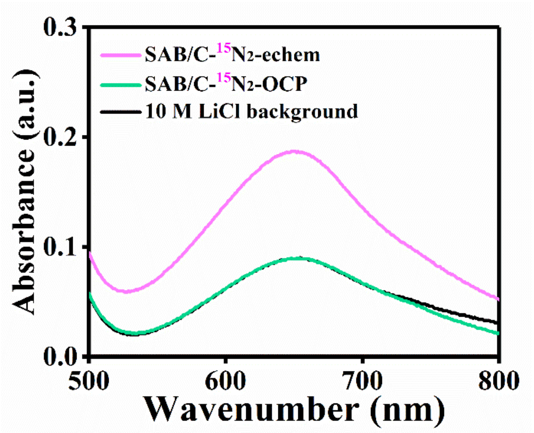


**Supplementary Figure 32.** UV-vis absorption spectra of the electrolytes under different conditions.

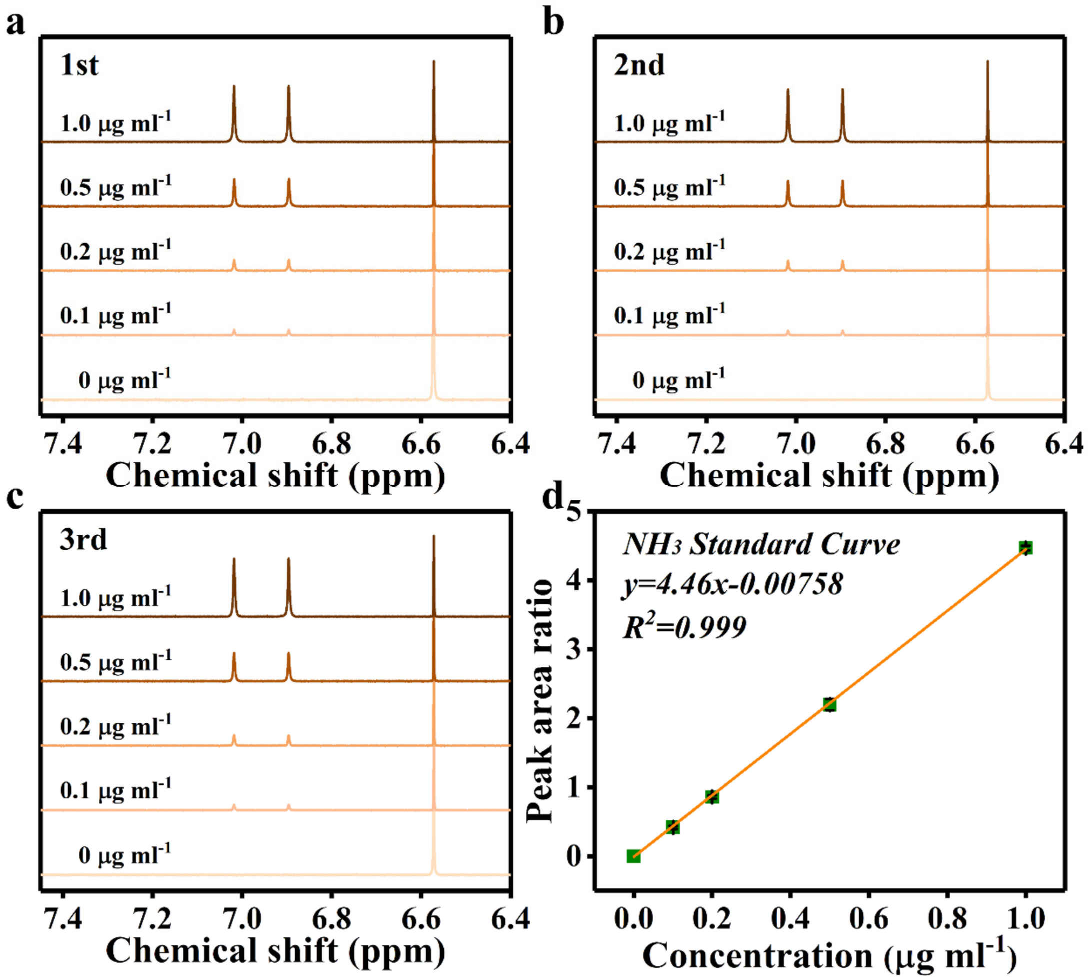


**Supplementary Figure 33. a-c** $^1$H NMR spectra of $^{15}NH_4^+$ with different normal concentrations from all three independent tests and **d** the corresponding standard curve for $^{15}NH_3$ concentration *vs.* peak area ratio. The error bars correspond to the standard deviations of measurements over three separately prepared samples under the same conditions. The calibration plots for the data points with error bars are almost perfectly linear, with an $R^2$ value of 0.999.

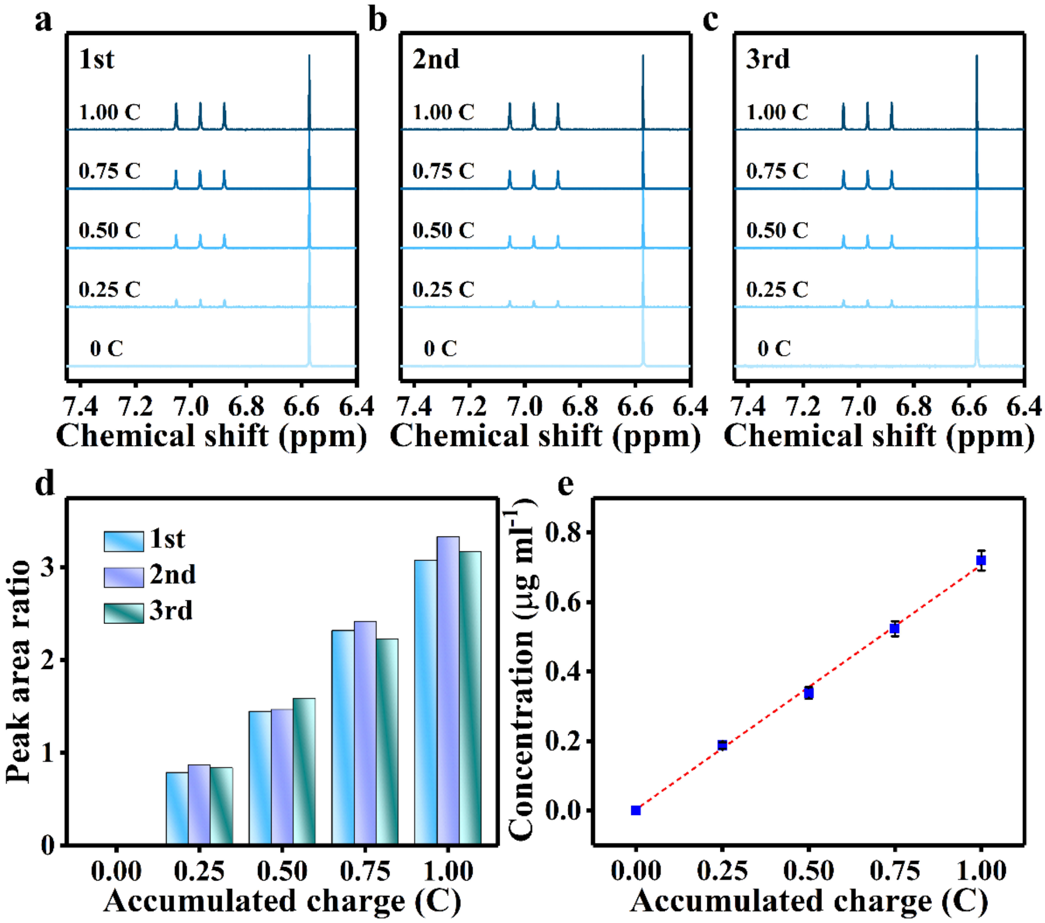


**Supplementary Figure 34. a-c** $^{1}H$ NMR spectra of $^{14}NH_4^+$ from all three $^{14}N_2$ reduction experiments as a function of charge passed. **d** The integral area ratio ($^{14}NH_4^+/C_4H_4O_4$) from all three independent tests as a function of charge passed. **e** The concentration of $^{14}NH_3$ as measured by NMR from the $^{14}N_2$ reduction experiments as a function of charge passed. The error bars correspond to the standard deviations of measurements over three separately prepared samples under the same conditions.

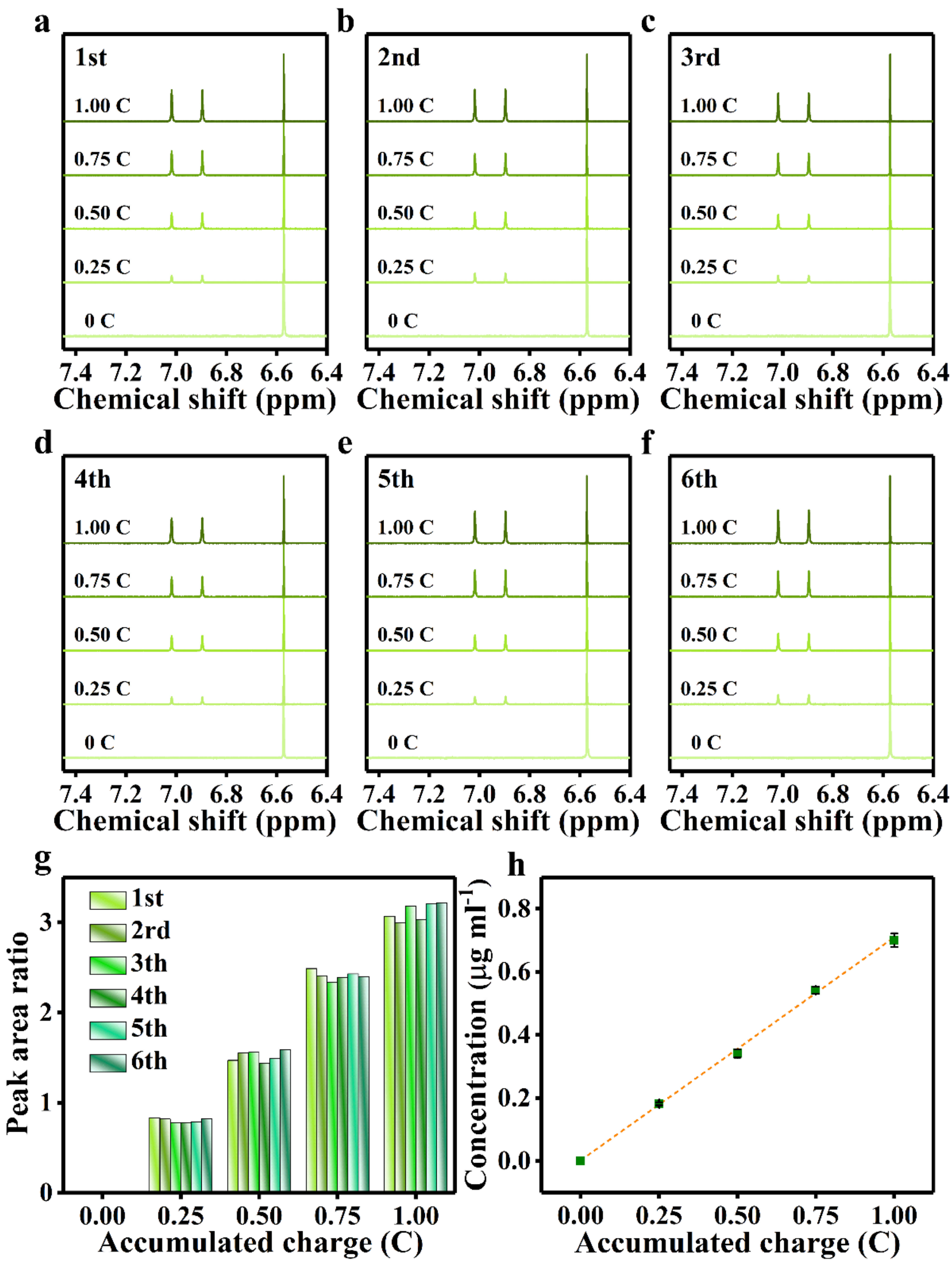


**Supplementary Figure 35. a-f** $^{1}H$ NMR spectra of $^{15}NH_4^+$ from all six $^{15}N_2$ reduction experiments as a function of charge passed. **g** The integral area ratio ($^{15}NH_4^+/C_4H_4O_4$) from all six independent tests as a function of charge passed. **h** The concentration of $^{15}NH_3$ as measured by NMR from the $^{15}N_2$ reduction experiments as a function of charge passed. The error bars correspond to the standard deviations of measurements over six separately prepared samples under the same conditions. Notably, although the $^{15}N_2$ gas supply contains no more than 1 atom % $^{14}N$, the concentration of the produced $^{14}NH_3$ is too low at even 1 C, which is below the limit of detection of the NMR method. Therefore, there is no triplet for $^{14}NH_4^+$ showing up in the $^{15}NH_4^+$ NMR spectra.

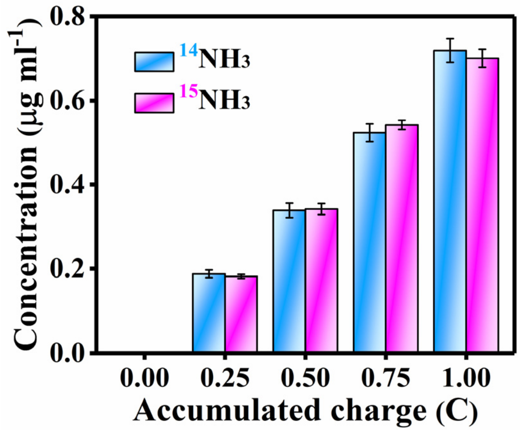


**Supplementary Figure 36.** Comparison of the concentration of $^{14}NH_3$ and $^{15}NH_3$, as measured by NMR, from the $^{14}N_2$ and $^{15}N_2$ reduction experiments, respectively, as a function of charge passed. The error bars correspond to the standard deviations of measurements over three ($^{14}N_2$) or six ($^{15}N_2$) separately prepared samples under the same conditions.

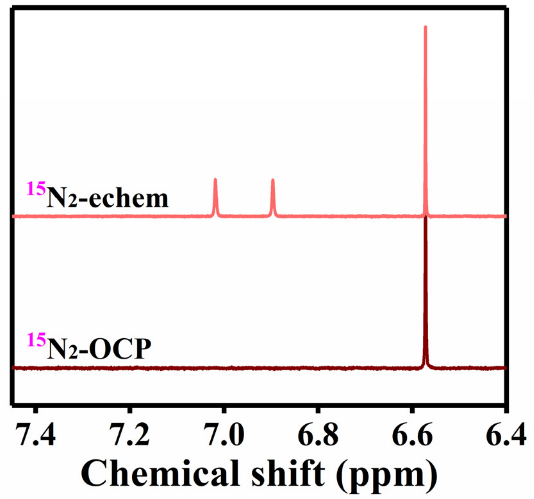


**Supplementary Figure 37.** NMR spectra of 10 M LiCl electrolyte after electrolysis with $^{15}N_2$ as the feeding gas at OCP and –0.3 V vs. RHE, respectively.

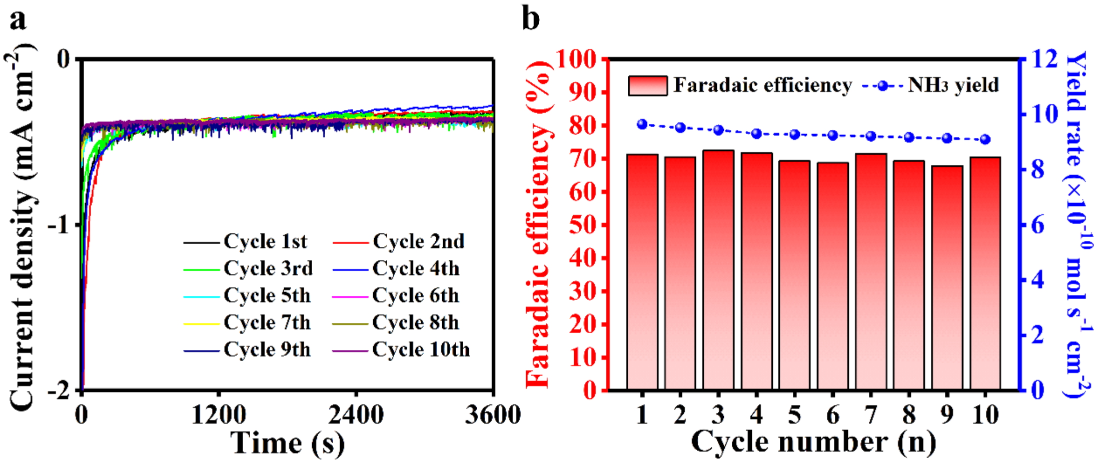


**Supplementary Figure 38. a** Chronoamperometry tests and **b** the NRR performance in durability test of 10 M LiCl.

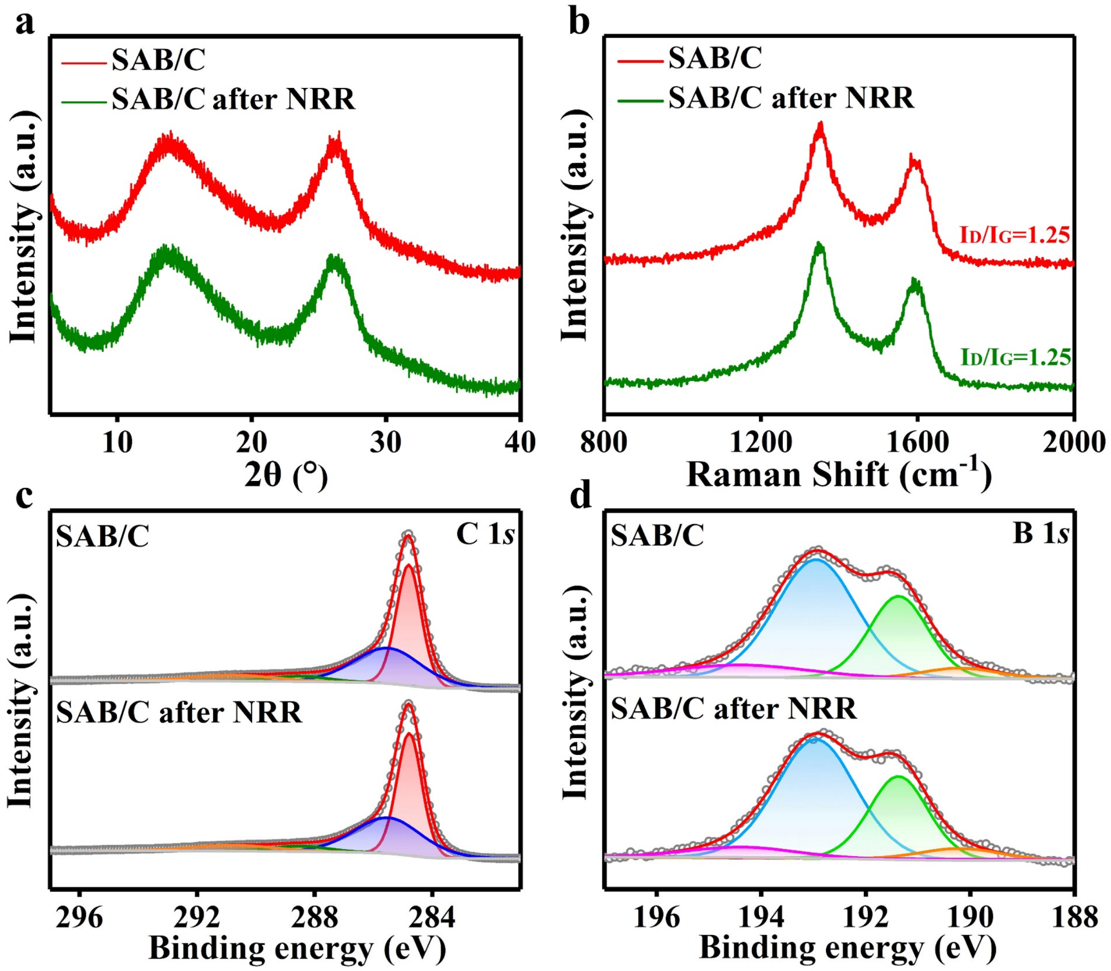


**Supplementary Figure 39.** **a** XRD patterns, **b** Raman spectra, high resolution **c** C 1*s* and **d** B 1*s* spectra of SAB/C before and after NRR electrolysis. The physical characteristics of the catalyst remain unchanged after the durability tests.

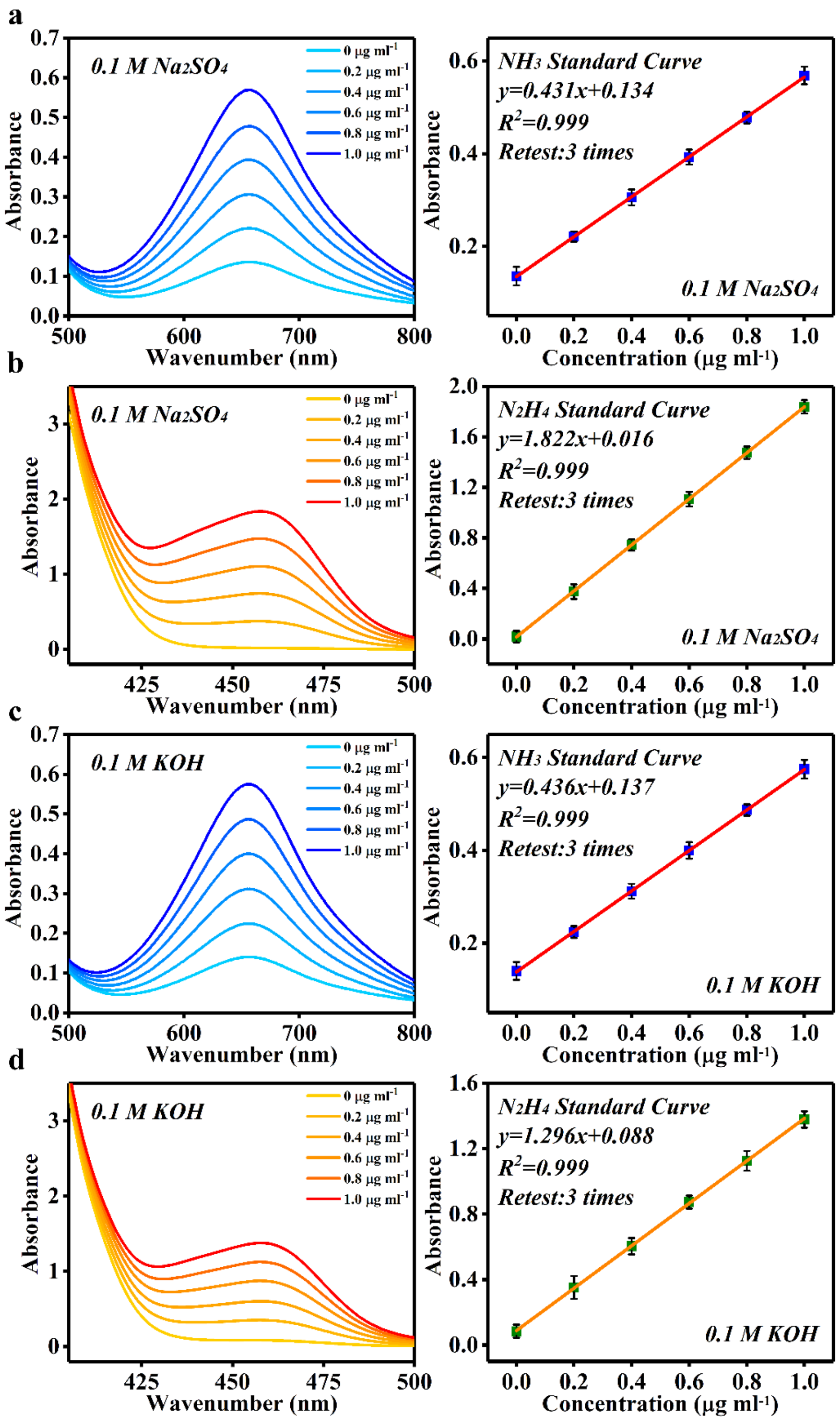


**Supplementary Figure 40.** Calibration curves for the colorimetric $NH_3$ assay using the indophenol blue method and $N_2H_4$ assay using the Watt and Chrisp method in **a**-**b** 0.1 M $Na_2SO_4$ and **c**-**d** 0.1 M KOH. The error bars correspond to the standard deviations of measurements over three separately prepared samples under the same conditions.

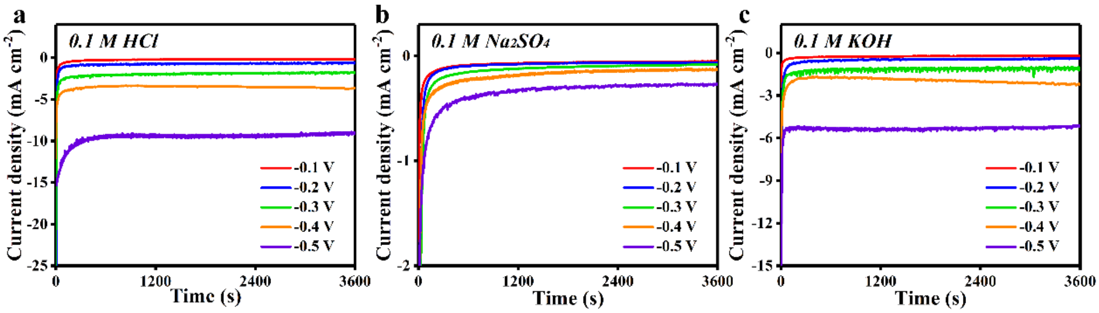


**Supplementary Figure 41.** Chronoamperometry results at the corresponding potentials in **a** 0.1 M HCl, **b** 0.1 M $Na_2SO_4$, and **c** 0.1 M KOH.

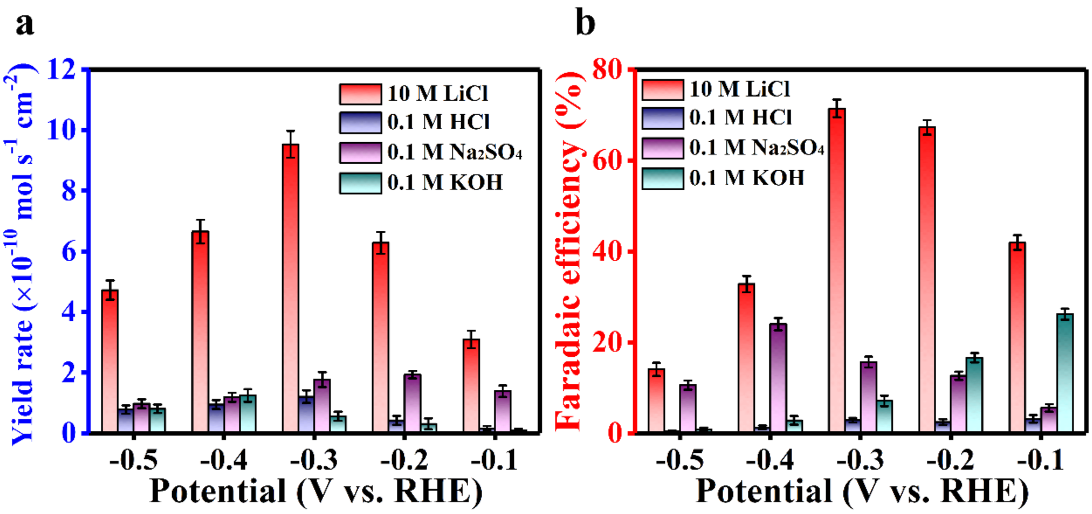


**Supplementary Figure 42.** Comparison of **a** $NH_3$ yield rates and **b** Faradaic efficiencies obtained in different electrolytes. The error bars correspond to the standard deviations of measurements over three separately prepared samples under the same conditions.

**Supplementary Table 1.** Interaction energy of ion-water, water-water, and gas-water within the heterogeneous catalysis system obtained by duplicate computations.

| Electrolyte | $E(Li^+-H_2O)/N(H_2O)$ ($kJ\ mol^{-1}$) | | $E(Cl^--H_2O)/N(H_2O)$ ($kJ\ mol^{-1}$) | | $E(N_2-H_2O)/N(H_2O)$ ($kJ\ mol^{-1}$) | | $E(H_2O-H_2O)/N(H_2O)$ ($kJ\ mol^{-1}$) | |
|---|---|---|---|---|---|---|---|---|
| | Mean value | Standard deviation | Mean value | Standard deviation | Mean value | Standard deviation | Mean value | Standard deviation |
| $H_2O$ | / | / | / | / | -0.18910 | 0.00198 | -46.31454 | 0.00603 |
| 2 M LiCl | -21.90765 | 0.03171 | -15.73369 | 0.01929 | -0.16728 | 0.01240 | -29.85641 | 0.02037 |
| 4 M LiCl | -38.45219 | 0.05013 | -26.54135 | 0.08594 | -0.11312 | 0.01674 | -18.31969 | 0.06276 |
| 6 M LiCl | -52.07439 | 0.12144 | -34.66213 | 0.08472 | -0.10653 | 0.00982 | -9.36866 | 0.08841 |
| 8 M LiCl | -62.43177 | 0.11279 | -40.09047 | 0.04254 | -0.10680 | 0.01259 | -3.05812 | 0.06563 |
| 10 M LiCl | -70.25339 | 0.30072 | -43.56459 | 0.15539 | -0.10766 | 0.00793 | 1.34229 | 0.16986 |
| 12 M LiCl | -75.47151 | 0.17342 | -45.24022 | 0.11898 | -0.11107 | 0.00458 | 3.87732 | 0.10771 |
| 14 M LiCl | -79.23216 | 0.34309 | -46.12786 | 0.06104 | -0.11529 | 0.00321 | 5.47179 | 0.12609 |

**Supplementary Table 2.** Water activity of the LiCl electrolytes with different concentrations.

| Electrolyte | Water activity |
|---|---|
| $H_2O$ | 0.990 |
| 2 M LiCl | 0.898 |
| 4 M LiCl | 0.793 |
| 6 M LiCl | 0.685 |
| 8 M LiCl | 0.551 |
| 10 M LiCl | 0.435 |
| 12 M LiCl | 0.316 |
| 14 M LiCl | 0.229 |

**Supplementary Table 3.** The $NH_3$ production amount (μg) in the catholyte (30 ml) and acid trap (30 ml) under different applied potentials.

| Electrolyte | | -0.1 V | -0.2 V | -0.3 V | -0.4 V | -0.5 V |
|---|---|---|---|---|---|---|
| 2 M LiCl | Catholyte | 1.802 | 4.876 | 3.074 | 0.318 | 0.212 |
| | Acid trap | 1.308 | 1.635 | 1.144 | 1.880 | 1.308 |
| 4 M LiCl | Catholyte | 0.745 | 2.979 | 0.745 | 0.638 | 0.532 |
| | Acid trap | 1.308 | 2.207 | 2.779 | 1.390 | 0.899 |
| 6 M LiCl | Catholyte | 1.932 | 3.977 | 3.182 | 0.341 | 0.227 |
| | Acid trap | 0.163 | 0.899 | 0.245 | 0.981 | 0.409 |
| 8 M LiCl | Catholyte | 0.798 | 4.563 | 9.125 | 2.281 | 2.281 |
| | Acid trap | 2.207 | 2.289 | 0.572 | 3.270 | 1.962 |
| 10 M LiCl | Catholyte | 1.875 | 8.555 | 11.484 | 9.609 | 5.508 |
| | Acid trap | 2.861 | 1.063 | 3.270 | 0.572 | 1.717 |
| 12 M LiCl | Catholyte | 1.528 | 1.944 | 6.389 | 5.833 | 4.722 |
| | Acid trap | 1.308 | 1.962 | 2.125 | 2.125 | 3.025 |
| 14 M LiCl | Catholyte | 0.484 | 0.645 | 1.452 | 3.387 | 5.806 |
| | Acid trap | 0.654 | 0.899 | 0.572 | 0.490 | 0.163 |

**Supplementary Table 4.** Comparison of the NRR performance of our work with the state-of-the-arts under ambient conditions (room temperature and atmospheric pressure).

| Catalyst | Electrolyte | Mass loading (mg $cm^{-2}$) | Faradaic efficiency (%) | $NH_3$ yield rate (mol $s^{-1}$ $cm^{-2}$) | $NH_3$ yield rate (mol $s^{-1}$ $mg^{-1}$) | Ref. |
|---|---|---|---|---|---|---|
| **Transition metal-based electrocatalysts** | | | | | | |
| Fe/fluorine doped tin oxide glass | Ionic liquids | 0.14 | 60 | $4.72\times10^{-12}$ | $3.33\times10^{-11}$ | 1 |
| VN | Nafion | 0.5 | 5.95 | $3.30\times10^{-10}$ | $6.60\times10^{-10}$ | 2 |
| MXene loaded on stainless steel mesh | 0.5 M $Li_2SO_4$ | 1.76 | 4.62 | $7.71\times10^{-11}$ | $4.38\times10^{-11}$ | 3 |
| Fe−N/C−carbon nanotubes | 0.1 M KOH | 0.5 | 9.28 | $2.85\times10^{-10}$ | $5.69\times10^{-10}$ | 4 |
| 2D $W_2N_3$ with nitrogen vacancies | 0.1 M KOH | 0.2 | 11.67 | $3.80\times10^{-11}$ | $1.90\times10^{-10}$ | 5 |
| Isolated single Fe atomic sites anchored to N-doped carbon frameworks | 0.1 M PBS | 1 | 18.6 | $1.02\times10^{-9}$ | $1.02\times10^{-9}$ | 6 |
| Single-atom dispersed Fe–N–C | 0.1 M KOH | 1 | 56.55 | $1.22\times10^{-10}$ | $1.22\times10^{-10}$ | 7 |
| **Metal-free electrocatalysts** | | | | | | |
| Poly(N-ethyl-benzene-1,2,4,5-tetracarboxylic diimide/C | 0.5 M $Li_2SO_4$ | 1.28 | 2.85 | $2.58\times10^{-11}$ | $2.02\times10^{-11}$ | 8 |
| $B_4C$ | 0.1 M HCl | 0.1 | 15.95 | $4.34\times10^{-11}$ | $4.34\times10^{-10}$ | 9 |
| N-doped porous carbon-750 | 0.05 M $H_2SO_4$ | 0.6 | 1.42 | $2.33\times10^{-10}$ | $3.89\times10^{-10}$ | 10 |
| Polymeric carbon nitride-nitrogen vacancies-4 | 0.1 M HCl | 2 | 11.59 | $2.65\times10^{-10}$ | $1.32\times10^{-10}$ | 11 |
| Alfalfa-derived N-doped porous graphitic carbon | 0.005 M $H_2SO_4$ | 1 | 9.98 | $3.64\times10^{-10}$ | $3.64\times10^{-10}$ | 12 |
| Boron-doped graphene | 0.05 M $H_2SO_4$ | 0.18 | 10.8 | $1.60\times10^{-10}$ | $8.50\times10^{-10}$ | 13 |
| SAB/C | 10 M LiCl | 1 | 71. ± 1.9 | $(9.5 \pm 0.4)\times10^{-10}$ | $(9.5 \pm 0.4)\times10^{-10}$ | This work |

**Supplementary Table 5.** Values of Lennard-Jones and Coulombic interaction potential parameters. Notably, for $N_2$ molecule, a point charge of +0.964 *e* was placed at the center of mass (COM) to maintain charge neutrality.

| Atom/ion | σ (Å) | ε (kJ mol$^{-1}$) | Charge (*e*) |
|---|---|---|---|
| H (in $H_2O$) | 0 | 0 | −0.8476 |
| O (in $H_2O$) | 3.169 | 0.6502 | +0.4238 |
| N (in $N_2$) | 3.31 | 0.2993 | –0.482 |
| COM (in $N_2$) | 0 | 0 | +0.964 |
| $Li^+$ | 1.505 | 0.6904 | +1.0 |
| $Cl^-$ | 4.401 | 0.4184 | –1.0 |

**Supplementary Table 6.** Purity of the $^{14}N_2$ gas supply purchased from Messer Gas Product Co. Ltd. (Germany).

| Component | Concentration |
|---|---|
| $^{14}N_2$ | $\geq$ 99.999% |
| $H_2$ | $\leq$ 1.0 ppm |
| $O_2$ | $\leq$ 3.0 ppm |
| $CH_4$ | $\leq$ 1.0 ppm |
| CO | $\leq$ 1.0 ppm |
| $CO_2$ | $\leq$ 1.0 ppm |
| $H_2O$ | $\leq$ 3.0 ppm |

**Supplementary Table 7.** Gas volumes used in each electrochemical experiment.

| Figure | Method | Electrolyte | Feed gas | Volume of gas used |
|---|---|---|---|---|
| Supplementary Figure 22 | Chronoamperometry | 2 M LiCl<br>4 M LiCl<br>6 M LiCl<br>8 M LiCl<br>10 M LiCl<br>12 M LiCl<br>14 M LiCl | $^{14}N_2$ | 2700 ml |
| Supplementary Figure 30 | | 10 M LiCl | $^{15}N_2$ | 300 ml |
| Supplementary Figure 33 | | 10 M LiCl | $^{14}N_2$ | 6300 ml |
| Supplementary Figure 34 | | 10 M LiCl | $^{15}N_2$ | 300 ml |
| Supplementary Figure 37 | | 10 M LiCl | $^{14}N_2$ | 2700 ml |
| Supplementary Figure 40 | | 0.1 M HCl<br>0.1 M $Na_2SO_4$<br>0.1 M KOH | $^{14}N_2$ | 2700 ml |

**Supplementary Table 8.** Limit of detection of colorimetric method and NMR method.

| Analytical method | Substance | Electrolyte | Limit of detection (ng ml$^{-1}$) |
|---|---|---|---|
| Indophenol blue method | $NH_3$ | $H_2O$ | 5.99 |
| | | 2 M LiCl | 10.01 |
| | | 4 M LiCl | 9.05 |
| | | 6 M LiCl | 10.08 |
| | | 8 M LiCl | 8.68 |
| | | 10 M LiCl | 9.67 |
| | | 12 M LiCl | 10.47 |
| | | 14 M LiCl | 13.44 |
| | | 0.1 M HCl | 7.78 |
| | | 0.1 M $Na_2SO_4$ | 6.23 |
| | | 0.1 M KOH | 5.86 |
| Watt and Chrisp method | $N_2H_4$ | 2 M LiCl | 2.22 |
| | | 4 M LiCl | 3.17 |
| | | 6 M LiCl | 3.17 |
| | | 8 M LiCl | 3.03 |
| | | 10 M LiCl | 3.66 |
| | | 12 M LiCl | 3.71 |
| | | 14 M LiCl | 5.33 |
| | | 0.1 M HCl | 5.13 |
| | | 0.1 M $Na_2SO_4$ | 2.19 |
| | | 0.1 M KOH | 3.22 |
| N-(-1-naphthyl)-ethylenediamine dihydrochloride spectrophotometric method | $NO_x$ | $H_2O$ | 16.87 |
| NMR method | $^{14}NH_3$ | 10 M LiCl | 39.46 |
| | $^{15}NH_3$ | 10 M LiCl | 45.57 |

**Supplementary Table 9.** Concentration of potential $NH_3$, $NO_x$, and $N_2O$ contaminants supplied in 12-h experiments using different feed gas and the corresponding maximum impurity contribution that could be expected.

| Gas | Flow rate (sccm) | Purging time (min) | Volume of gas used (ml) | $NH_3$[#] (ng ml$^{-1}$) | $NO_x$[#] (ng ml$^{-1}$) | $N_2O$ (ppm) | Maximum impurity contribution (mol s$^{-1}$) |
|---|---|---|---|---|---|---|---|
| **Commercial $^{14}N_2$** | **30** | **720** | **21600** | **5.99*** | **136.90** | **0.05*** | **$4.54\times10^{-12}$** |
| **Purified $^{14}N_2$** | | | | **5.99*** | **16.87*** | **0.05*** | **$2.73\times10^{-12}$** |
| **Commercial $^{15}N_2$** | **0** | **30 (prior to Echem at 10 sccm)** | **300** | **5.99*** | **23.81** | **0.79** | **$1.09\times10^{-12}$** |
| **Purified $^{15}N_2$** | | | | **5.99*** | **16.87*** | **0.76** | **$0.97\times10^{-12}$** |

[#]The concentrations of $NH_3$ and $NO_x$ were based on 30 ml electrolyte.

*For the contaminants that were not detected, their respective concentration was assumed to be equal to the corresponding limit of detection.

**References**

1. Zhou, F. L., Azofra, L. M., Ali, M., Kar, M., Simonov, A. N., McDonnell-Worth, C. M., Sun, C. H., Zhang, X. Y. & MacFarlane, D. R. Electro-synthesis of ammonia from nitrogen at ambient temperature and pressure in ionic liquids. *Energy Environ. Sci.* **10**, 2516−2520 (2017).

2. Yang, X. et al. Mechanistic insights into electrochemical nitrogen reduction reaction on vanadium nitride nanoparticles. *J. Am. Chem. Soc.* **140**, 13387−13391 (2018).

3. Luo, Y., Chen, G. –F., Ding, L., Chen, X., Ding, L. –X. & Wang, H. Efficient electrocatalytic $N_2$ fixation with MXene under ambient conditions. *Joule* **3**, 1–11 (2019).

4. Wang, Y. et al. Rational design of Fe−N/C hybrid for enhanced nitrogen reduction electrocatalysis under ambient conditions in aqueous solution. *ACS Catal.* **9**, 336–344 (2019).

5. Jin, H., Li, L., Liu, X., Tang, C., Xu, W., Chen, S., Song, L., Zheng, Y. & Qiao, S. −Z. Nitrogen vacancies on 2D layered $W_2N_3$: A stable and efficient active site for nitrogen reduction reaction. *Adv. Mater.* **31**, 1902709 (2019).

6. Lü, F. et al. Nitrogen-coordinated single Fe sites for efficient electrocatalytic $N_2$ fixation in neutral media. *Nano Energy* **61**, 420−427 (2019).

7. Wang, M., Liu, S., Qian, T., Liu, J., Zhou, J., Ji, H., Xiong, J., Zhong, J. & Yan, C. Over 56.55% Faradaic efficiency of ambient ammonia synthesis enabled by positively shifting the reaction potential. *Nat. Commun.* **10**, 341 (2019).

8. Chen, G. –F. et al. Ammonia electrosynthesis with high selectivity under ambient conditions via a $Li^+$ incorporation strategy. *J. Am. Chem. Soc.* **139**, 9771–9774 (2017).

9. Qiu, W. et al. High-performance artificial nitrogen fixation at ambient conditions using a metal-free electrocatalyst. *Nat. Commun.* **9**, 3485 (2018).

10. Liu, Y. et al. J. Facile ammonia synthesis from electrocatalytic $N_2$ reduction under ambient conditions on N-doped porous carbon. *ACS Catal.* **8**, 1186−1191 (2018).

11. Lv, C. et al. Defect engineering metal-free polymeric carbon nitride electrocatalyst for effective nitrogen fixation under ambient conditions. *Angew. Chem. Int. Ed.* **57**, 10246–10250 (2018).

12. Zhao, C. et al. Ambient electrosynthesis of ammonia on a biomass-derived nitrogen-doped porous carbon electrocatalyst: Contribution of pyridinic nitrogen. *ACS Energy Lett.* **4**, 377−383 (2019).

13. Yu, X., Han, P., Wei, Z., Huang, L., Gu, Z., Peng, S., Ma, J. & Zheng, G. Boron-doped graphene for electrocatalytic $N_2$ reduction. *Joule* **2**, 1–13 (2018).
