# Supplementary material for: Salting-out effect promoting highly efficient ambient ammonia synthesis
Source: Nat Commun. 2021 May 27;12:3198. doi: 10.1038/s41467-021-23360-0 (PMC8160333; doi:10.1038/s41467-021-23360-0)
Supplement: Supplementary file 2 — Description of Additional Supplementary Files [file 41467_2021_23360_MOESM2_ESM.docx]

**Description of Additional Supplementary Files**

File Name: Dataset 1

Description: Parameters of the catalyst model for molecular dynamics simulations.

File Name: Dataset 2

Description: NMR and electrochemical measurements
